# Supplementary material for: Label-free mass and size characterization of few-kDa biomolecules by hierarchical vision transformer augmented nanofluidic scattering microscopy
Source: Nat Commun. 2026 Mar 13;17:2533. doi: 10.1038/s41467-026-70514-z (PMC12996530; doi:10.1038/s41467-026-70514-z)
Supplement: Supplementary file 1 — Supplementary Information [file 41467_2026_70514_MOESM1_ESM.pdf]

Supplementary Information - Label-Free Mass and Size Characterization of Few-kDa Biomolecules by Hierarchical Vision Transformer Augmented Nanofluidic Scattering Microscopy

Henrik K. Moberg[1], Bohdan Yeroshenko[1], Joachim Fritzsche[1], David Albinsson[2], Barbora Spackova[3], Daniel Midtvedt[4], Giovanni Volpe[4] & Christoph Langhammer[1,*]

[1]Department of Physics, Chalmers University of Technology, Sweden
[2]Envue Technologies, Sweden
[3]Institute of Physics of the Czech Academy of Sciences, Czech Republic
[4]Department of Physics, Gothenburg University, Sweden

[*]To whom correspondence should be addressed; E-mail: clangham@chalmers.se.

1 Molecular Property Characterization

Determining the MW of a biomolecule from its trajectory inside a nanofluidic channel is possible because the integrated optical contrast (iOC) of said trajectory in the kymograph is linearly dependent on the polarizability α_m of the biomolecule [1], where iOC is defined as

$$\text{iOC} = \int_{x=0}^{L} (I_t(x) - I_c(x))/I_c(x)dx \tag{1}$$

for a channel of length L. The polarizability α_m is in turn proportional to $a \times$ MW, where $a \approx 0.46$ Å^3/Da [1]. Furthermore, the iOC is inversely proportional to the cross-sectional area A of the nanochannel and proportional to $\bar{n} = \left(1.5n^2_{H_2O} + 0.5n^2_{SiO_2}\right) / \left(n^2_{H_2O} - n^2_{SiO_2}\right)$ [2]. The former means that nanochannels with smaller cross-section boost the iOC and is the reason why we use smaller channels for our Insulin experiments, and why BSA becomes distinctly visible in channel A_{II}. Since both these factors are constant and easily measured before an experiment, the

MW of a molecule measured by NSM can be determined as

$$\mathrm{MW} = (\mathrm{iOC} \cdot A)/(\bar{n}a). \tag{2}$$

Determining the R_s of a biomolecule from its trajectory is possible because its diffusivity D is correlated with its size. In particular, by approximating the molecule as a solid neutrally charged sphere, its R_s can be estimated using the Stokes-Einstein equation with a correction term K for hindrance effects arising from the diffusion of small objects in restricted volumes [3], as we have demonstrated in our seminal work[2]. R_s is estimated as

$$R_\mathrm{s} = K k_B T/(6\pi\eta D), \tag{3}$$

where k_B is Boltzmann's constant, T is temperature, and η is the viscosity of the liquid in the nanochannel.

2 Deep Learning Architecture

Neural networks, including deep learning architectures such as the Hierarchical Vision Transformer (h-ViT) employed in this work, are fundamentally piecewise linear function approximators capable of representing any function through the partitioning of hyperplanes in the input space during training. The depth of a neural network determines the complexity of these partitions, thereby enhancing the representational power of the architecture. In the case of h-ViT for NSM, subsequent layers progressively refine the latent representation of the input data, allowing for the identification of hyperplanar partitions that best approximate molecular weight and hydrodynamic radius of biomolecules inside nanofluidic channels.

A key advantage of deep learning, and specifically of the h-ViT model, is its ability to capture long-range dependencies in microscopy data through attention mechanisms and hierarchical feature extraction. Unlike conventional CNNs, which rely on local receptive fields and pooling layers to reduce dimensionality, h-ViT processes nanofluidic scattering kymographs using a transformer-based multi-scale approach. Each transformer block integrates multi-head self-attention, enabling the model to selectively focus on different regions of the input and thus improve the estimation of diffusivity and integrated optical contrast. This allows the h-ViT model to learn more physically relevant, generalizable, and interpretable features from microscopy images.

The limitations of traditional convolutional neural networks arise from their inductive biases, particularly their focus on spatial locality and translational invariance. While this is useful in conventional imaging tasks, microscopy data often requires global context awareness, which h-ViT achieves by replacing local receptive fields with attention-based mechanisms. The model encodes input kymographs using a custom PatchEncoder layer, which extracts feature-rich patches at multiple scales. These patches are then processed by transformer layers that retain spatial relationships across entire kymographs, allowing for superior feature extraction.

Traditional analytical approaches for microscopy often rely on explicit noise modeling and handcrafted filters, which limit their effectiveness in complex, non-stationary

noise environments. In contrast, deep learning approaches like h-ViT leverage spectral bias to extract relevant low-frequency information while maintaining sensitivity to high-frequency molecular motion. This is particularly advantageous in denoising diffraction-limited images, where low-frequency trends hidden within high-frequency noise patterns can be effectively recovered.

In Nanofluidic Scattering Microscopy, where single molecules diffuse inside nanofluidic channels, standard CNN-based methods fail to fully capture the underlying molecular properties due to low signal-to-noise ratios (SNR) and the inherently stochastic nature of molecular motion. h-ViT addresses these challenges by employing probability maps that quantify the likelihood of molecular presence in different regions of the kymograph. These probability maps serve as intermediate feature representations that guide the final prediction of MW and R_s.

The model refines its predictions through a weighted averaging mechanism, ensuring that high-confidence regions contribute more significantly to the final MW and R_s estimation. This probabilistic approach allows h-ViT to outperform classical CNN architectures in low-SNR microscopy settings, making it an essential tool for molecular characterization at the single-molecule level.

In applications such as label-free single-molecule microscopy, the h-ViT model has significantly improved the lower detection limits (LoD) of NSM, achieving molecular weight resolutions down to 6 kDa and hydrodynamic radius resolutions below 1.5 nm. This is a major advancement over conventional analytical approaches, which previously struggled to characterize molecules below 60 kDa and 4 nm. The improved LoD is attributed to a combination of ultrasmall nanochannel fabrication (29 nm $\times$ 60 nm cross-section) and the deep-learning-driven signal extraction capabilities of h-ViT.

Additionally, h-ViT enables real-time analysis of diffusing biomolecules without requiring prior surface immobilization, overcoming a major limitation of alternative methods such as interferometric scattering microscopy (iSCAT). The combination of hierarchical attention mechanisms and transformer-based feature learning allows h-ViT to robustly estimate MW and R_s even when traditional tracking algorithms fail due to low SNR.

The h-ViT model represents a fundamental shift in deep-learning-based microscopy, replacing conventional CNN-based approaches with hierarchical, transformer-based architectures that preserve global spatial relationships and efficiently extract biomolecular features. The integration of attention-driven, multi-scale processing within NSM enables significantly enhanced molecular characterization, setting a new benchmark for deep-learning-powered optical microscopy.

2.1 Hierarchical Vision Transformer

The deep learning architecture presented in this study, the Hierarchical Vision Transformer (h-ViT), enhances the characterization of biomolecules using NSM. It builds on recent advancements in attention mechanisms and transformer-based architectures to capture the multi-scale nature of kymograph data effectively. The input kymograph,

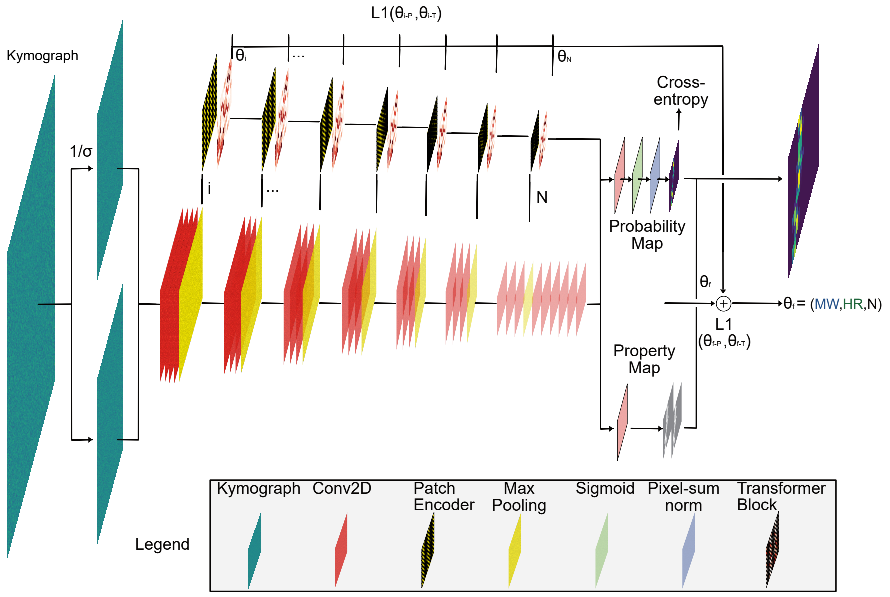

Supplementary Figure 1: **Hierarchical Vision Transformer (h-ViT) architecture for single biomolecule characterization by NSM.** The h-ViT model processes NSM kymographs to predict the MW and R_s of single particles, such as biomolecules. Initially, Conv2D layers extract spatial features, followed by max pooling for downsampling. The Patch Encoder then encodes these feature maps into smaller patches, while retaining crucial spatial information. Each patch is passed through Transformer Blocks, where multi-head attention mechanisms selectively focus on different regions of the input, capturing long-range dependencies in the kymograph. The outputs of the Transformer Blocks are processed to generate two key outputs: a Probability Map (indicating the likelihood of particle trajectories at different locations) and a Property Map (predicting specific biomolecule properties, such as MW and R_s). The model utilizes cross-entropy loss for classification tasks and L1 loss to minimize the error between predicted and true molecular properties. The estimation of each parameter (θ_i) representing MW and R_s, and number of trajectory points, N, is refined through iterative back-propagation. Finally, the model weighs the Property Map with the Probability Map to output a final estimation of the biomolecule properties, weighted by the probability of said biomolecule being present in any given region.

a two-dimensional representation of scattered light intensity over time, undergoes preprocessing through temporal standard deviation normalization to ensure consistency in subsequent feature extraction.

The kymograph is first processed through a convolutional layer with a kernel size of 7 and leaky ReLU activation to preserve subtle variations in the data. This is followed by a series of convolutional blocks that progressively downsample the data while capturing features at different spatial and temporal scales. The architecture employs a hierarchical multi-scale approach in which max-pooling operations reduce spatial dimensions at each stage. Encoded patches from feature maps are processed through transformer blocks that integrate multi-head attention mechanisms, allowing the model to selectively focus on different regions of the input. These transformer layers enhance the ability to capture long-range dependencies and refine molecular property estimations, such as diffusivity and iOC.

Instead of reconstructing explicit pixel-wise trajectories, h-ViT employs probability maps that encode the likelihood of particle presence at specific positions and times. This allows the model to aggregate information across the entire kymograph rather than being constrained by localized trajectory visibility, which is often compromised due to low signal-to-noise ratios. Multi-head attention mechanisms dynamically emphasize regions that correlate with iOC (and thus molecular weight, MW) and diffusivity (and thus hydrodynamic radius, R_s). This formulation ensures that the model extracts global patterns indicative of particle motion while mitigating noise amplification.

A key advantage of h-ViT is its mitigation of spectral bias, wherein deep learning models tend to prioritize low-frequency over high-frequency signals. In NSM kymographs, particle trajectories constitute low-frequency signals embedded within high-frequency noise. The hierarchical structure of h-ViT forces the model to extract relevant trends at different downsampling scales, improving sensitivity to weak scattering signals from small particles. Unlike conventional convolutional approaches, which often overfit to noisy pixel-wise variations, h-ViT prioritizes broader contextual features, thereby improving detection limits.

The model generates predictions for particle properties at multiple downsampling scales, which are processed through dense layers and probability maps to quantify the likelihood of particle presence. These probability maps are resized to match the original kymograph dimensions, ensuring spatial alignment with input data. The outputs from different scales are then concatenated to form a minimally dimensioned representation, condensing all relevant particle information across scales. The final predictions of MW, R_s, and the number of trajectory points (N) are computed using a weighted averaging mechanism, where higher-probability regions contribute more significantly to the output.

This hierarchical and attention-driven design marks a departure from traditional convolutional neural networks by emphasizing global feature extraction rather than local trajectory reconstruction. The ability to infer molecular properties from probability-weighted feature maps, rather than explicit trajectory tracing, significantly improves robustness in low-SNR conditions. As demonstrated in this study, the h-ViT model substantially enhances the sensitivity and accuracy of NSM-based biomolecule characterization, setting a new benchmark for deep-learning-driven optical microscopy.

Section	Item	Value	Notes
Global input	Raw input shape	$(20480, 512, 1)$	T=20480, L=512
Pre-norm fusion	Extra channel	$x/(\text{std}_{(T,L)} + 10^{-6})$	Concatenate with raw input ⇒ 2 channels
Initial stem	Conv 7×7	2 filters, LeakyReLU, BN	Param: 198 (conv) + 4 (BN) = 202
Downsampling depth	Number of scales	7	One 2×2 max-pool per scale
Pooling stride	Per scale	$(2, 2)$	Cumulative stride $2^{\text{scale}+1}$
Feature map sizes	After pooling (0→6)	See below	
Channels per scale	`num_filters`(s)	$2, 4, 8, 16, 32, 64, 128$	$2 \cdot 2^s$ (base = 2)
Patch size per scale	`patch_size_current`	$64, 32, 16, 8, 4, 2, 1$	$128/2^{s+1}$
Tokens per scale	# patches	$160 \times 4 = 640$ (all scales)	Constant sequence length
Patch embedding dim	d_{model}	128	Scale-specific encoders share spec
Patch projection params	Per scale (proj + pos emb)	See below	Sum = 2,655,104
Transformer depth	Blocks per scale	3	Total blocks = 21
Attention heads	# heads	8	
Multi-head attention	Key dimension (per head)	128	Attn width = 128×8=1024; output projection 1024→128 for residual match
Sequence length	Tokens	640	Uniform across scales
MLP in block	Hidden → output	128 → 128 (GELU, then linear to 128)	MLP Dimension = 128
Normalization	Per block	2× LayerNorm ($\varepsilon = 10^{-6}$)	Pre/post residual
Dropout	In block	Rrate = 0.1	Not applied to attention/MLP outputs
Probability heads (per scale)	Conv 3×3 → sigmoid	1 channel prior to further pooling	Params per scale: $9C + 1$

Section	Item	Value	Notes
Final probability fusion	Aggregation	Median across scales (channel axis)	No learnable parameters
Gated regression head	Mechanism	Conv(128) × final prob map → GAP → Dense(3)	Spatially weighted pooling
Per-scale property heads	Outputs	7× Dense(128→3) with LeakyReLU	
Fused scale head	Outputs	Dense(21→3)	Concatenate 7×(3)
Final regression head	Outputs	Dense(128→3)	After gated pooling
Output list (order)	Total outputs	7 per-scale (B,3), fused (B,3), final (B,3), final prob map (B,160,4,1)	10 outputs
Spatial resolution	Final map	$(160, 4)$	Effective stride 128×128
Effective receptive stride	Relative to input	128 in both axes	Implied by 7 pooling stages
**Total parameters**	**All modules**	**≈ 15,016,819**	
Conv front-end params	Stem + residual stacks	427,172	Includes pooling pathways
Transformer parameters	Self-attention blocks	11,781,504	21 blocks @ 561,024 each
Patch encoder parameters	Total	2,655,104	7 independent encoders
Head & map parameters	Total	153,039	Property heads + prob head + final conv/dense
Loss configuration	Objective	[MAE]×(7+2)+[BCE]	Matches 10 outputs
MAE targets	Components	MW (intensity), HR (size), trajectory length	Mean absolute error sums
BCE	Type	Weighted binary cross-entropy	Weight factor = 10 on final prob map
Activations	CNN & heads	LeakyReLU (sigmoid for masks)	
Computational strategy	Constant token length	Uniform transformer cost across scales	Multi-scale invariance

Section	Item	Value	Notes
Normalization strategy	Input & blocks	Input pre-norm channel, BN after stem conv, LayerNorm inside blocks	
Gating rationale	Mask × features	Suppress irrelevant regions prior to pooling	Robust regression

Parameter Summary

Component	Parameters
Convolutional front end (stem + 7 residual blocks + pooling)	427,172
Patch encoders (projection + positional embeddings)	2,655,104
Transformer blocks (21 × 561,024)	11,781,504
Probability map convolutions (7 scales)	2,293
Per-scale property heads (7 × 387)	2,709
Fused scale head	66
Final convolution (3×3, 128→128)	147,584
Final dense (128→3)	387
**Total**	≈ 15,016,819

Per-Scale Configuration

Scale	Spatial (H,W)	Channels	Patch size	Patch dim	Patch enc params	Block params (3 blocks)
0	10240 × 256	2	64	8192	1,130,624	1,683,072
1	5120 × 128	4	32	4096	606,336	1,683,072
2	2560 × 64	8	16	2048	344,192	1,683,072
3	1280 × 32	16	8	1024	213,120	1,683,072
4	640 × 16	32	4	512	147,584	1,683,072
5	320 × 8	64	2	256	114,816	1,683,072
6	160 × 4	128	1	128	98,432	1,683,072

3 Deep Learning Training

In this section, we delve deeper into the specific training methods which are vital to reaching the high accuracy and precision of prediction (and thus low NSM LoD) of

the trained models used. In particular, we describe transfer learning; the process of transferring learnt knowledge from one network to another, and curriculum learning; the process of successively increasing the difficulty of the task during training to be learnt to facilitate learning in very challenging conditions.

3.1 Transfer Learning

Transfer learning is a technique that involves transferring knowledge gained while solving one task to another (similar but not identical) task. The underlying idea is that some of the knowledge and skills learned while solving the first problem can be applied directly to the second problem, resulting in improved performance and shorter training time. It has been shown that a neural network trained on a particular type of dataset is much better at quickly recognizing new instances of similar data, when compared to a completely untrained network [4]. For instance, though a neural network may need thousands of examples of cars to start to accurately recognize cars, such a network can learn much quicker to recognize other vehicles when introduced into its training set compared to an untrained network [5]. This is a consequence of neural networks learning increasingly complex abstractions in deeper layers of the network. Thus, a majority of the learning a network has to do to solve any given task is simply to find a good representation of the data, and transfer learning can thus be achieved by "freezing" the training of the first layers of a neural network and only training the last few layers on the new data.

There are several benefits of this approach, most evidently that it can help reduce training time and data requirements for new tasks. Another benefit is that it can improve generalization performance, meaning that models trained using transfer learning are more likely to achieve good results on unseen data than those trained from scratch specifically for the new task at hand. Additionally, because transfer learning typically relies on pre-trained models which have been already tuned for good performance on a wide range of tasks, it can help avoid overfitting problems which can plague neural networks when they are retrained from scratch for a specific purpose.

Despite its many advantages, there are also some potential drawbacks to consider with transfer learning strategies. One issue may be poor adaptation if there are significant differences between how tasks were originally learned and how they need to be applied later on, and special care should be taken when applying pre-trained models so as to not inadvertently introduce bias into results due to inaccuracies or irregularities in how the old network was transfer-learned into the new.

3.2 Curriculum Learning

Human beings often do not learn anything when approached with an insurmountable challenge, learn only the minimally required information when approached with a trivial problem, and learn optimally only when approached with a problem perfectly tuned to be slightly challenging given their particular skillset [6]. Neural networks, interestingly, have been shown to learn in analogous ways [7]. To take advantage of this, we can implement active learning or curriculum learning [8] schemes, wherein we

vary which portions of the datasets are fed into the neural network during training to tune the challenges of learning, for instance by automatically feeding in more examples of data-points in regimes where the network retains low accuracy. Neural networks need to train on a large amount of data only to learn optimal representations of the dataset in its initial layers, but once these representations are learnt, transfer-learning it on new data is usually only a question of tuning the specifics of the last few layers' learnt abstractions. To take advantage of this, we might employ a curriculum learning scheme wherein we train the networks with the easiest examples or the ones we have the most data-points of first, and successively increase the difficulty of the dataset as the network's loss decreases.

Projected onto the challenges at hand in the present work, to train the molecular weight- and hydrodynamic radius-calculating h-ViT model, a curriculum learning scheme with intermittent checkpoints to be used for later ensemble modelling prediction was employed. Specifically, the model was initially trained only on a narrow range of high MW trajectories, representing the highest SNR and in principle easiest case for the model to begin learning correlations, and then slowly curriculum-learned down to the lowest range of relevant MW values. The process is equivalent for diffusivity, with the difference being that the range of D values being trained on increases rather than decreases during curriculum learning.

To ensure optimal training progression, the molecular weight (MW) was initially sampled from the range 50-200 kDa and progressively reduced in steps of 5% down to a final range of 0-30 kDa. This allowed the model to first learn from larger, well-defined particles before adapting to the more challenging detection of smaller biomolecules with lower signal contrast. Similarly, the diffusivity (D) was initially sampled from 10-50 $\mu m^2/s$ and progressively increased in steps of 5% up to 50-300 $\mu m^2/s$. This gradual expansion of diffusivity ensured that the model first learned from slower-moving particles, where trajectory tracking is more reliable, before adapting to highly diffusive molecules, which require more robust inference techniques. The values are updated after the loss has converged, with a patience of 100 epochs. This curriculum learning approach systematically expanded the model's training distribution, improving its ability to generalize across a broad range of biomolecular properties.

3.3 Training Configuration and Hyperparameters

The h-ViT model was trained using the Adam optimizer with an initial learning rate of 10^{-4}, employing an AMSGrad variant to improve convergence stability. A batch size of 8 was used, ensuring sufficient generalization while maintaining computational efficiency. Weight decay was set to 5×10^{-2} to prevent overfitting, and gradient clipping was applied at 1.0 to stabilize training. The transformer layers incorporated a dropout rate of 0.1 to mitigate over-reliance on specific activation paths. Early stopping was applied with a patience of 100 epochs based on the total loss.

The loss function for training h-ViT consisted of multiple components reflecting the hierarchical nature of the architecture. At each of the seven hierarchical scales, the model predicted molecular weight (MW), hydrodynamic radius (R_{s}), and the number

of trajectory points (N). The loss for these predictions was computed as the mean absolute error (MAE), ensuring that deviations from true values were minimized in a robust manner. The total loss across all scales was formulated as:

$$\mathcal{L}_{\text{hierarchical}} = \sum_{s=1}^{7} \left(\text{MAE}(MW_s^{\text{pred}}, MW_s^{\text{true}}) + \text{MAE}(R_{s,s}^{\text{pred}}, R_{s,s}^{\text{true}}) + \text{MAE}(N_s^{\text{pred}}, N_s^{\text{true}}) \right) . \tag{4}$$

In addition to hierarchical loss, the model also predicted a probability map that quantifies the likelihood of particle presence at specific spatiotemporal positions. This probability map was supervised using a binary cross-entropy loss, ensuring that the predicted spatial localization of biomolecules aligned with their true positions:

$$\mathcal{L}_{\text{mask}} = -\sum_{i,j} \left(y_{i,j} \log \hat{y}_{i,j} + (1 - y_{i,j}) \log(1 - \hat{y}_{i,j}) \right) , \tag{5}$$

where $y_{i,j}$ represents the ground truth probability of a particle at position (i, j) in the kymograph, and $\hat{y}_{i,j}$ is the model's predicted probability.

The final loss function combined the hierarchical loss with the probability map supervision:

$$\mathcal{L} = \mathcal{L}_{\text{hierarchical}} + \lambda_{\text{mask}} \mathcal{L}_{\text{mask}}, \tag{6}$$

where $\lambda_{\text{mask}} = 10$ was used to ensure adequate weight for the probability map supervision in relation to the hierarchical property estimation.

Following training, the h-ViT model was validated on an independent dataset of experimental kymographs. The model demonstrated high accuracy, achieving molecular weight predictions within 5% of the ground truth for $MW < 10$ kDa for higher trajectory lengths. Most notably, the model successfully reached molecular weight detection limits down to 6 kDa, approaching the theoretical lower bound for NSM-based detection as defined by the CRLB.

4 Generative Adversarial Network for Trajectory Generation

Image segmentation is used to make the value of each pixel of a given image more representative of a property of interest. In our case, we transform a kymograph where the value of each pixel represents intensity, to a segmented image where the value of each pixel represents the probability P_b of the existence of a biomolecule in that location and time. This transformation is achieved using a U-net, through the DeepTrack 2.0 Python software package [9]. The U-net's structure is illustrated in Supplementary Figure 2. The U-net's training was integrated into a conditional Generative Adversarial Network (GAN). This GAN is composed of two neural networks (depicted in

Supplementary Figure 7B): the generator network (our U-net), which produces image segmentations from input kymographs, and a discriminator network, which tries to discern if the generated segmentations truly represent the trajectories of individual biomolecules in the input kymographs. Throughout the training phase, these networks challenge each other, leading the generator to produce increasingly authentic trajectories, while the discriminator refines its ability to differentiate between real and generated trajectories. Utilizing this GAN for training was crucial to attain the necessary precision, particularly for tinier biomolecules, where the weak signal-to-noise ratio can cause their trajectories to be completely masked by noise across consecutive time frames.

The U-net's training was based on simulated kymographs (created and pre-processed as outlined in 'Methods'), for which the exact trajectory of a single biomolecule is established. The training employed the ADAM optimizer [10] with a learning rate that decays exponentially at 109, a decay rate set at 0.9, and spanning 50 decay steps. The U-net was trained on 300,000 simulated kymographs with particle trajectories within the bounds $1e-9$ μm $\leq$ iOC $\leq$ $3e-3$ μm $1 \leq D \leq 100\mu m^2/s$. The training data comprised simulated images (kymographs), containing a variable number of trajectories, and corresponding segmented images of the same size. The model underwent validation every 120 epochs (equivalent to approximately 30,000 simulated kymographs) against 150 simulated kymographs that incorporated experimentally observed channel noise, following an 80-20 train-validation distribution.

5 Simulated dataset

The data used to generate figures 3 and 4 consists of $n_{samples} \cdot n_{trajectories} \cdot n_{\mathrm{MW}} = 12 \cdot 11 \cdot 20 = 2640$ simulated kymographs, with $n_samples = 24$ number of samples per trajectory length and MW, for 11 trajectory lengths logarithmically equally spaced between $100 - 20480$ frames and MW $\in [1.5, 3, 5, 7, 9, 14, 28]$ kDa. The R_{s} of each simulated trajectory is calculated assuming the molecule is a globular protein diffusing through a (constraining) nano-channel, using the phenomenological model introduced in [3] to correct for hindrance effects related to the impact of the nanochannels' constrictions on the diffusivity of the molecules. Specifically, the resulting R_{s} of the simulated particles is $R_{\mathrm{s}} = [1, 1.3, 1.5, 1.8, 2.1, 2.6]$ nm.

5.1 h-ViT as a Biased Estimator

As a key point, the h-ViT performs as a biased estimator dependant on the estimated molecular trajectories derived from its generated probability maps. Here, we show alternative visualizations of the underlying results, focusing on molecular weight and hydrodynamic radius predictions derived from our experiments, to further demonstrate this effect.

These visualizations highlight the robustness of our predictive framework, as longer trajectory lengths reduce uncertainties in both molecular weight and hydrodynamic radius estimations. The systematic improvement with increasing L underscores the importance of trajectory length in enhancing predictive accuracy. Specifically, we

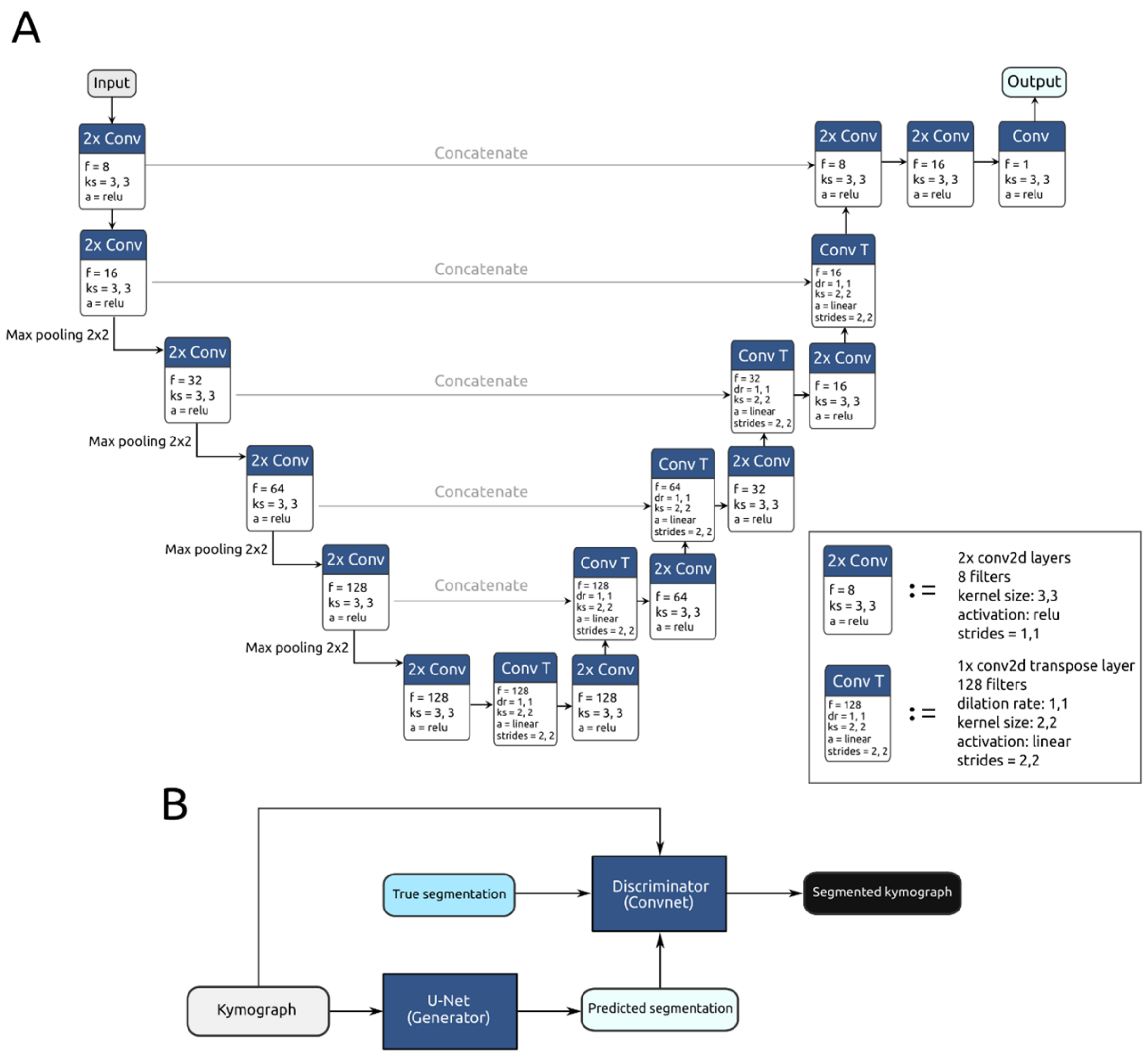

Supplementary Figure 2: U-net for single biomolecule segmentation. (A) The U-net [11] consists of a series of contraction convolutional layers, a bottleneck, and a series of expansion convolutional layers, as well as a series of skip connections between corresponding contraction and expansion convolutional layers to ensure that information learnt during contraction is not lost at the bottleneck. Each 2x Conv box represents a convolutional block corresponding to 2 convolutional layers in sequence, and each Conv T box represents a single convolutional transpose layer as exemplified in the legend. Here, f is the number of filters in each block, ks is the kernel size, a is the activation function, and dr is the dilation rate. The network is visualized with Netron [12]. (B) Block diagram of the GAN training environment, where kymographs are fed both to a U-net generator network, which predicts a corresponding segmentation, and a Convnet (convolutional network) discriminator network, which takes both original kymographs and true segmentations as input to determine whether the predicted segmentation is correct. A basic Convnet consisting of 5 convolutional layers of size 4x4 and stride 1 connected to a single dense layer is used as discriminator network [13].

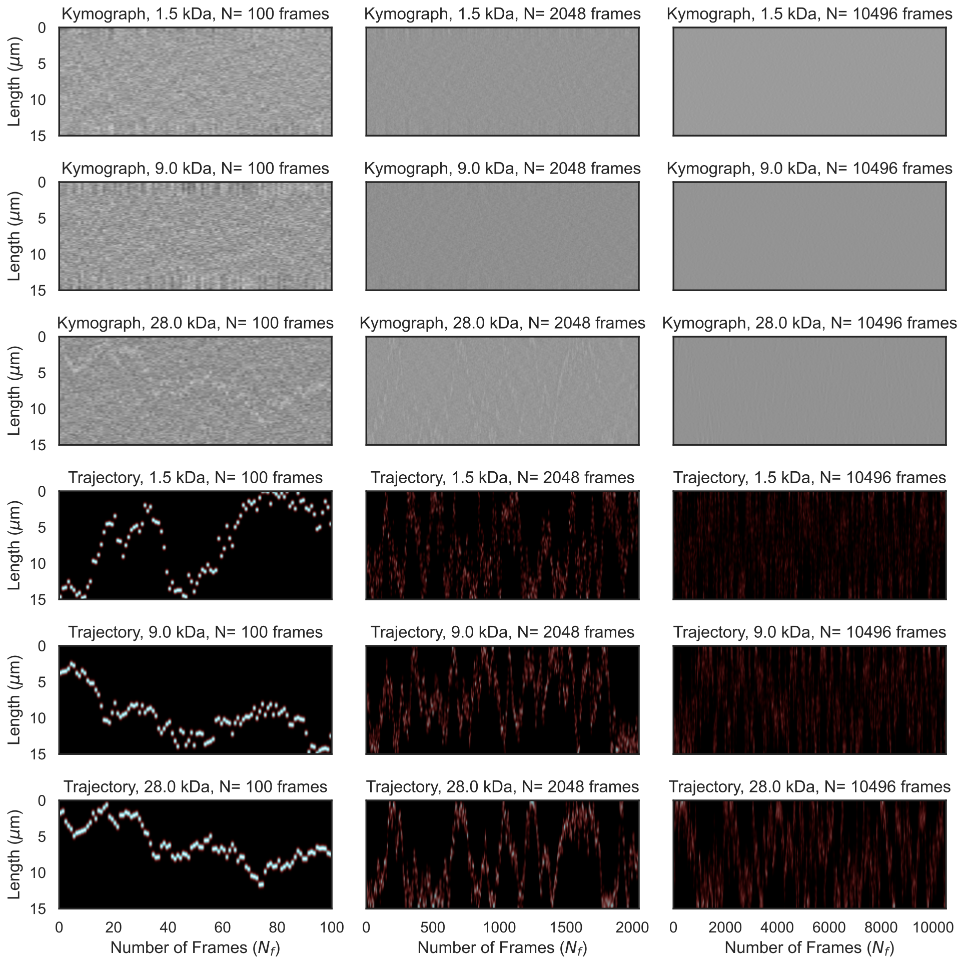

Supplementary Figure 3: Representative subset of simulated dataset, consisting of simulated kymographs for $MW \in [1.5, 9, 28]$ kDa in channel $A_I I$, and trajectory lengths $L \in [100, 2048, 10496]$

observe that for shorter trajectory lengths, the model's predictions exhibit higher variance, reflecting limited sampling and lower statistical confidence. As trajectory lengths approach 10,000 frames, the predictions converge towards the true molecular weight and hydrodynamic radius values, with residual errors diminishing significantly. Conversely, the precision increases as the accuracy decreaes for shorter trajectory lengths.

This at first somewhat surprising result can be understood as a consequence of the model being a biased estimator which tends to underfit the data at intermediate trajectory lengths. This bias occurs because the model is designed to interpret kymographs

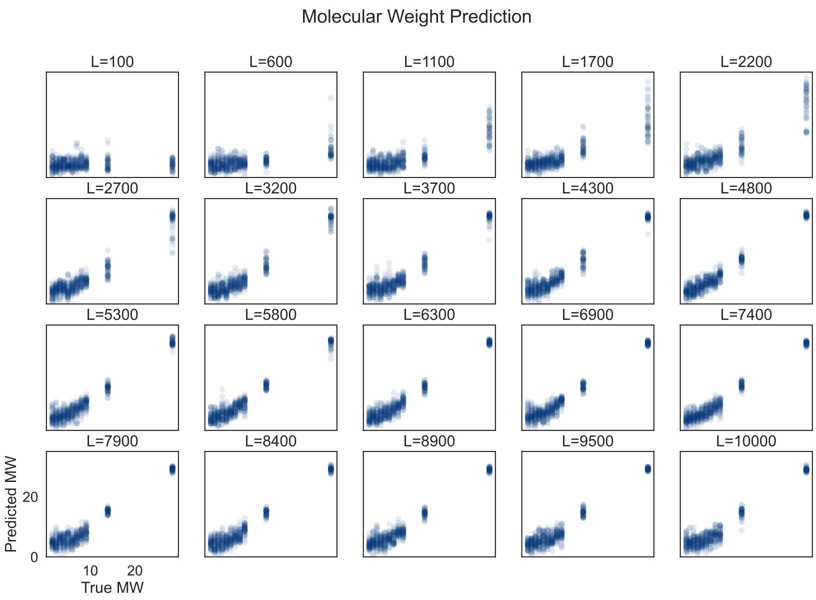

Supplementary Figure 4: Predictions underlying the results in Figure 2 of the main text, consisting of predicting molecular weight of simulated molecules for $MW \in [10, 20]$ kDa for reference channel Ch_B for trajectory lengths L between 100 and 10,000 frames. True molecular weights are plotted against predicted molecular weights across a range of trajectory lengths, illustrating the convergence of the predictions with increasing L.

with small N as highly likely to be noise, resulting in a systematic tendency to predict MWs close to zero and R_s close to 1 nm, in these cases, which for small true MW and R_s values indeed is a quite "good" estimate. As N increases, the network has more data at its disposal, allowing it to increasingly distinguish between actual signal due to the presence of a particle and noise. Nevertheless, initially in this regime, the precision of the model prediction decreases because the network is no longer dismissing as much data as noise. Hence, it starts to process kymographs that, for smaller N, would have been classified as noise and thus assigned MWs close to zero or R_s values close to 1 nm. Consequently, this introduces transiently larger variance into the predictions, which is reflected in the observed transiently reduced precision. However, with even further increasing N, the model continues to refine its predictions, as the additional trajectory points provide clearer particle signals, and both accuracy and precision improve. By the time the trajectory length reaches $N = 10000$, the h-ViT model is thus able to accurately and consistently predict MW and R_s of the 6 kDa particle, thereby effectively balancing the trade-off between filtering out noise and accurately capturing signals in the kymogrpah that stem from the presence of a particle.

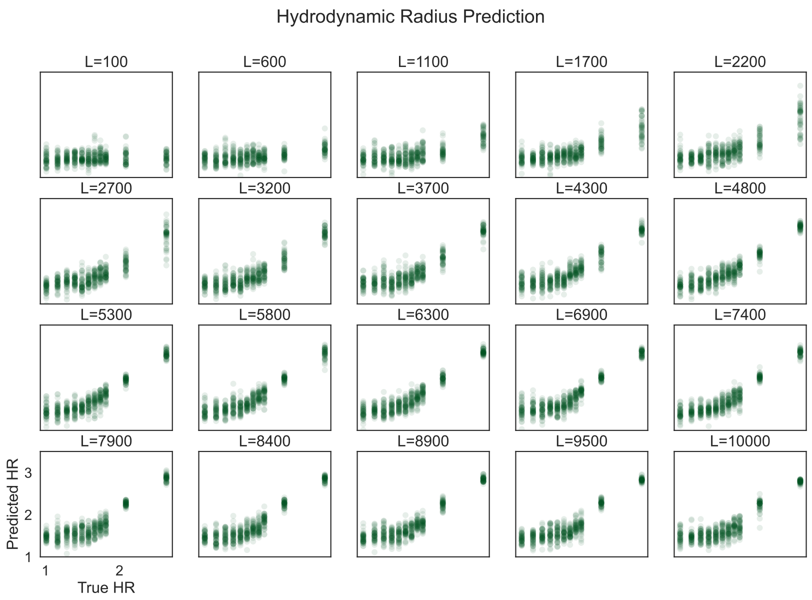

Supplementary Figure 5: Predictions underlying the results in Figure 2 of the main text, consisting of predicting hydrodynamic radius of simulated molecules for $MW \in [10, 20]$ kDa for reference channel Ch_B for trajectory lengths L between 100 and 10,000 frames. True hydrodynamic radii are plotted against predicted radii, showing improvement in prediction accuracy with longer trajectory lengths.

The statistical underpinnings of these trends warrant further discussion. For molecular weight predictions, the error decay aligns with a $1/\sqrt{L}$ dependence, consistent with central limit theorem principles applied to stochastic sampling. This suggests that as more frames are included, the effective sample size increases, enhancing the signal-to-noise ratio and reducing random fluctuations in the measurements. Similarly, hydrodynamic radius predictions benefit from extended trajectories through improved temporal averaging, which smooths transient deviations arising from molecular diffusion and interaction dynamics.

The convergence behavior also reflects the interplay between model fidelity and data sufficiency. For $L < 1000$, the predictions are influenced more strongly by prior assumptions and the model's intrinsic limitations, as data scarcity imposes constraints on predictive precision. Beyond this threshold, the influence of empirical data grows dominant, enabling the model to capture subtle molecular variations with high accuracy. This transition underscores the dual necessity of robust modeling frameworks and sufficient experimental data to achieve reliable characterization.

In addition, these findings have broader implications for experimental design and methodological optimization. The demonstrated improvement with longer trajectories emphasizes the need for high-throughput, long-duration measurements in

nanochannel-based systems to fully exploit their analytical potential. Future studies could explore adaptive sampling strategies that dynamically adjust trajectory lengths based on real-time prediction confidence, thereby balancing measurement efficiency and accuracy. This approach could further enhance the applicability of nanochannel-based techniques across diverse fields, including polymer science, biophysics, and molecular diagnostics.

6 Full DNA Measurements

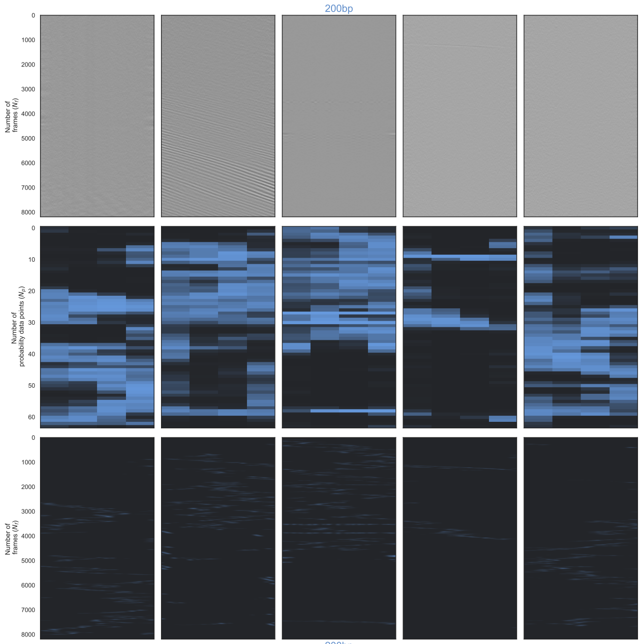
200bp
Number of frames (N_f)
0
1000
2000
3000
4000
5000
6000
7000
8000
Number of probability data points (N_p)
0
10
20
30
40
50
60
Number of frames (N_f)
0
1000
2000
3000
4000
5000
6000
7000
8000

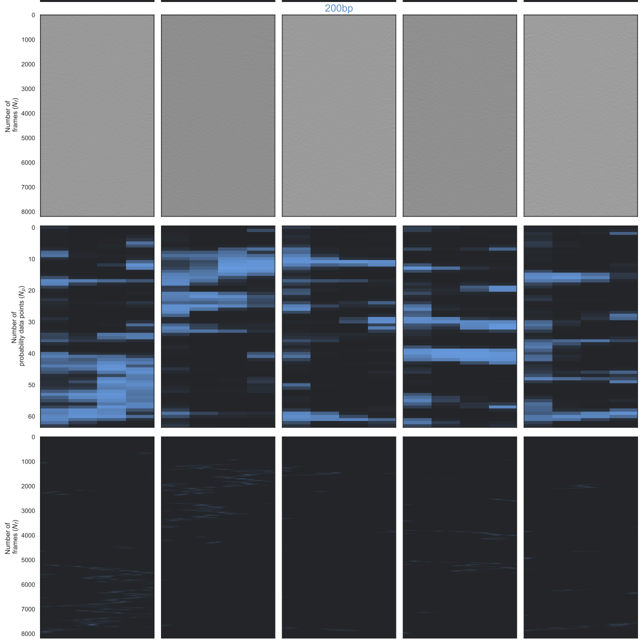
200bp
Number of frames (N_f)
0
1000
2000
3000
4000
5000
6000
7000
8000
Number of probability data points (N_p)
0
10
20
30
40
50
60
Number of frames (N_f)
0
1000
2000
3000
4000
5000
6000
7000
8000

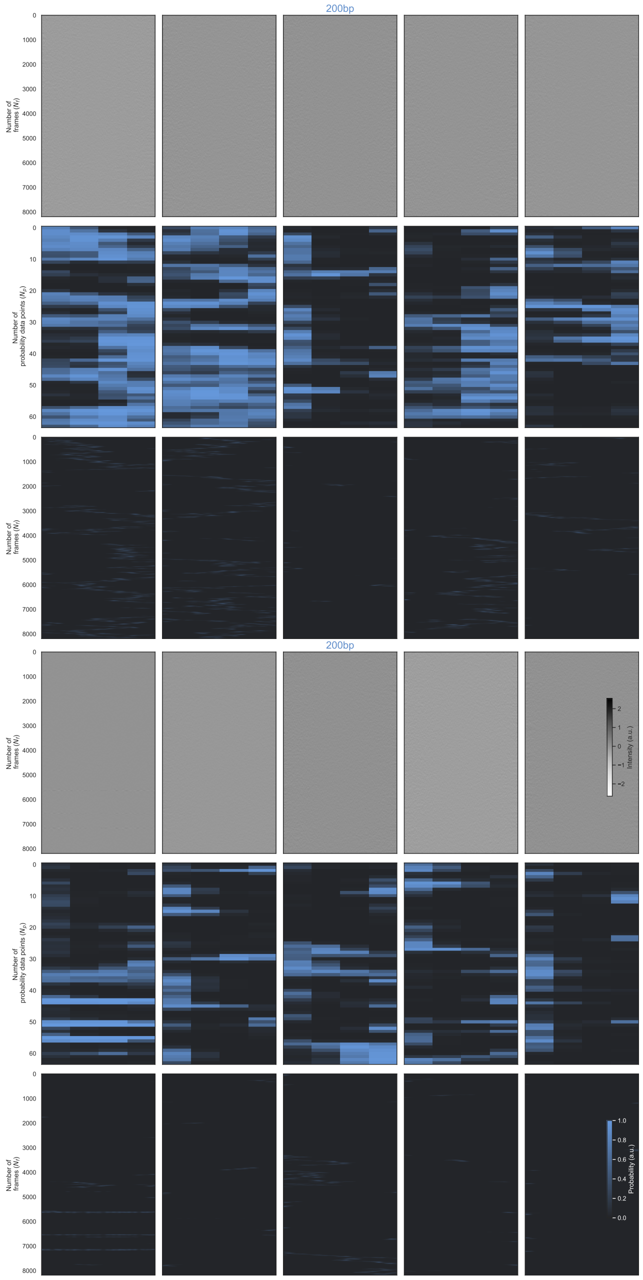

Supplementary Figure 7: **DNA Measurements in a Nanofluidic Channel with 122 nm x 97 nm cross sectional dimension.** The experimental results of analyzing , and of a pure TE-buffer control are shown. (**A**) Experimentally measured NSM kymographs for 200 bp double-stranded DNA fragments in TE-buffer. (**B**) Probability maps generated by the h-ViT model using the kymograps in (A) as input. (**C**) Segmented kymographs generated by the cGAN model using the kymograps in (A) as input.

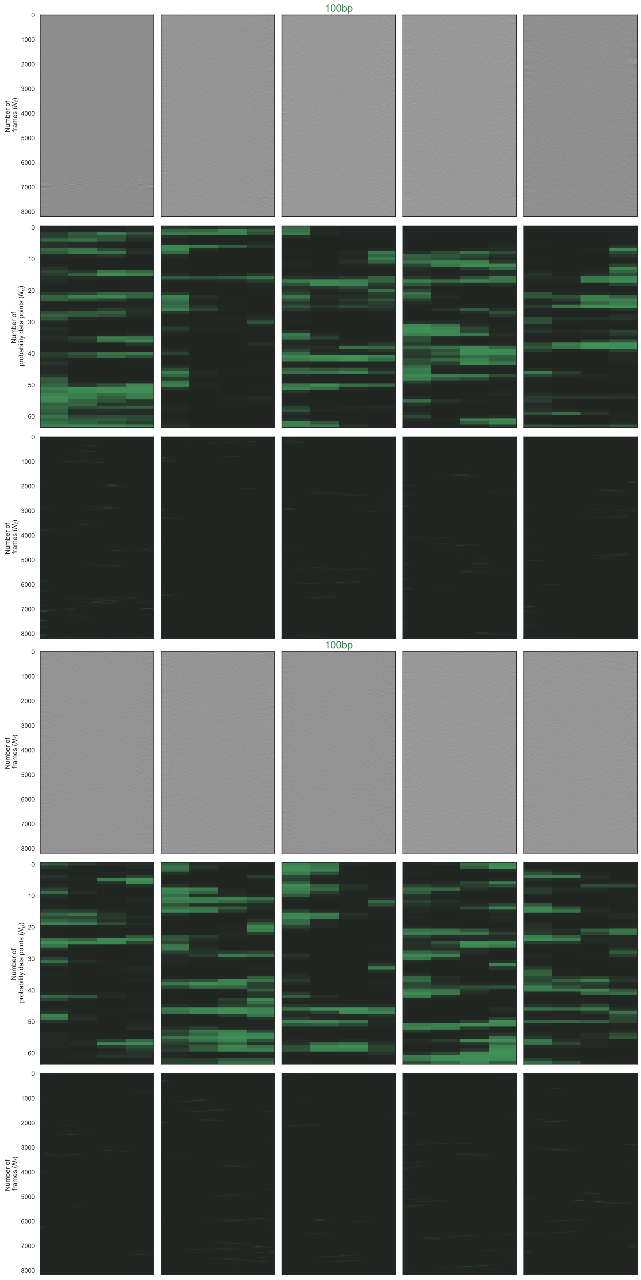
100bp
Number of frames (Nf)
Number of probability data points (Np)
100bp
Number of frames (Nf)
Number of probability data points (Np)

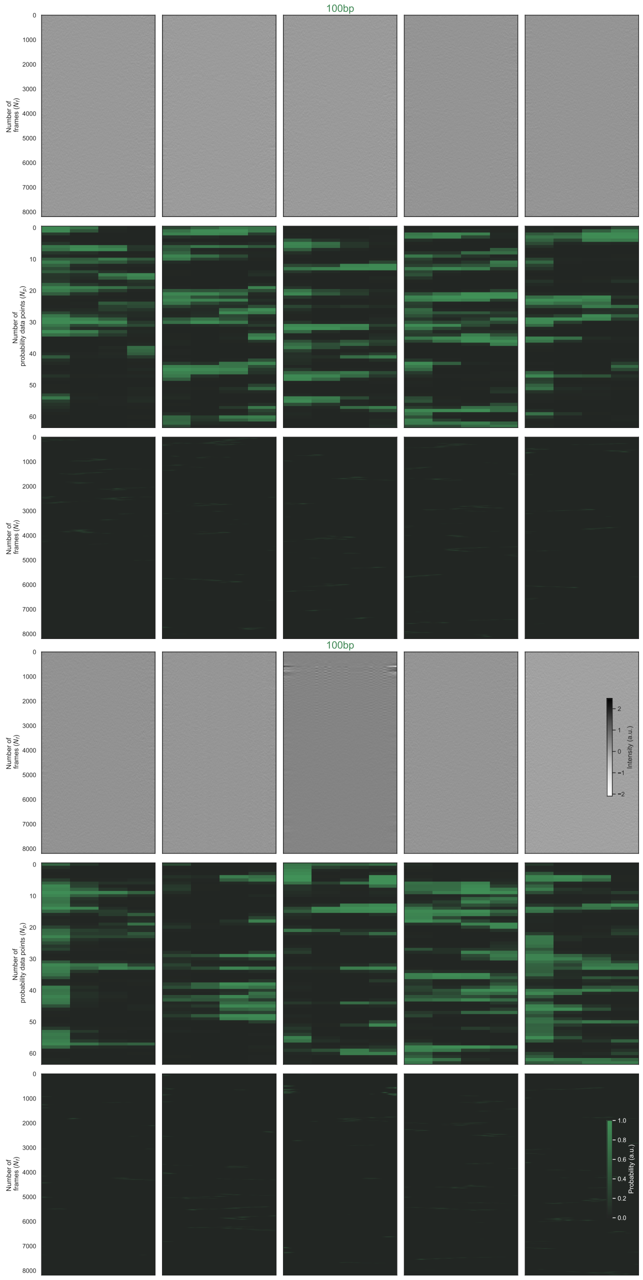

Supplementary Figure 9: Equivalent to Supplementary Figure 7, but for 100 bp double stranded DNA.

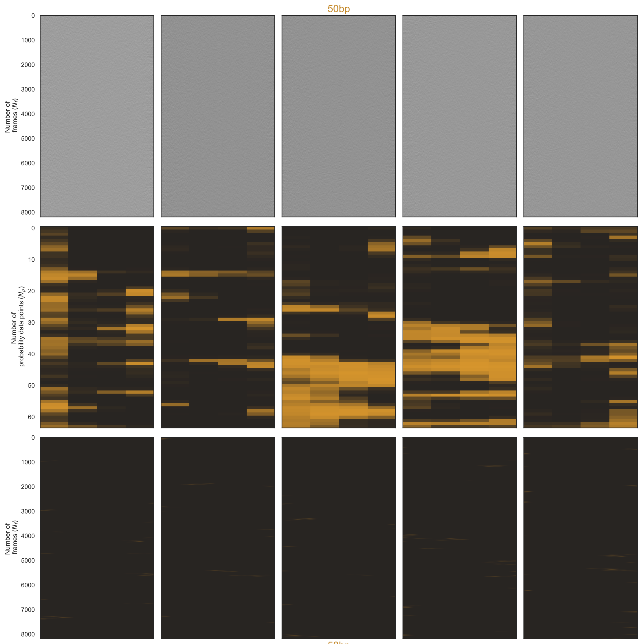
50bp
Number of frames (N_f)
Number of probability data points (N_p)
Number of frames (N_f)

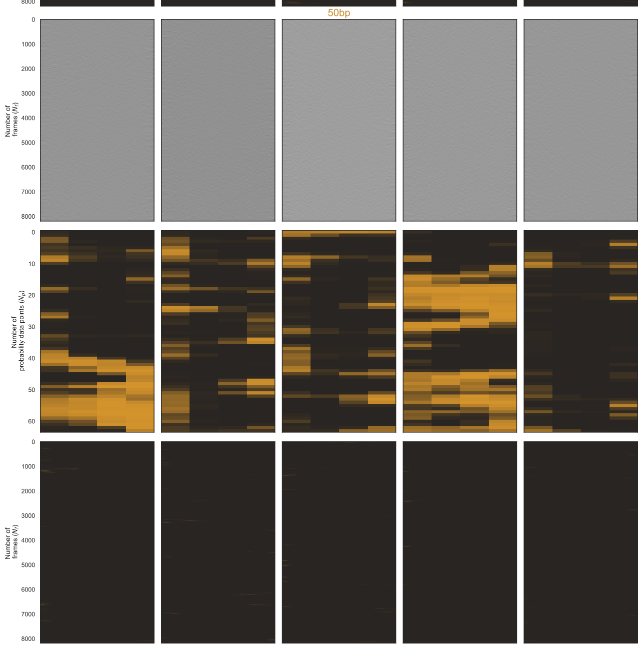
50bp
Number of frames (N_f)
Number of probability data points (N_p)
Number of frames (N_f)

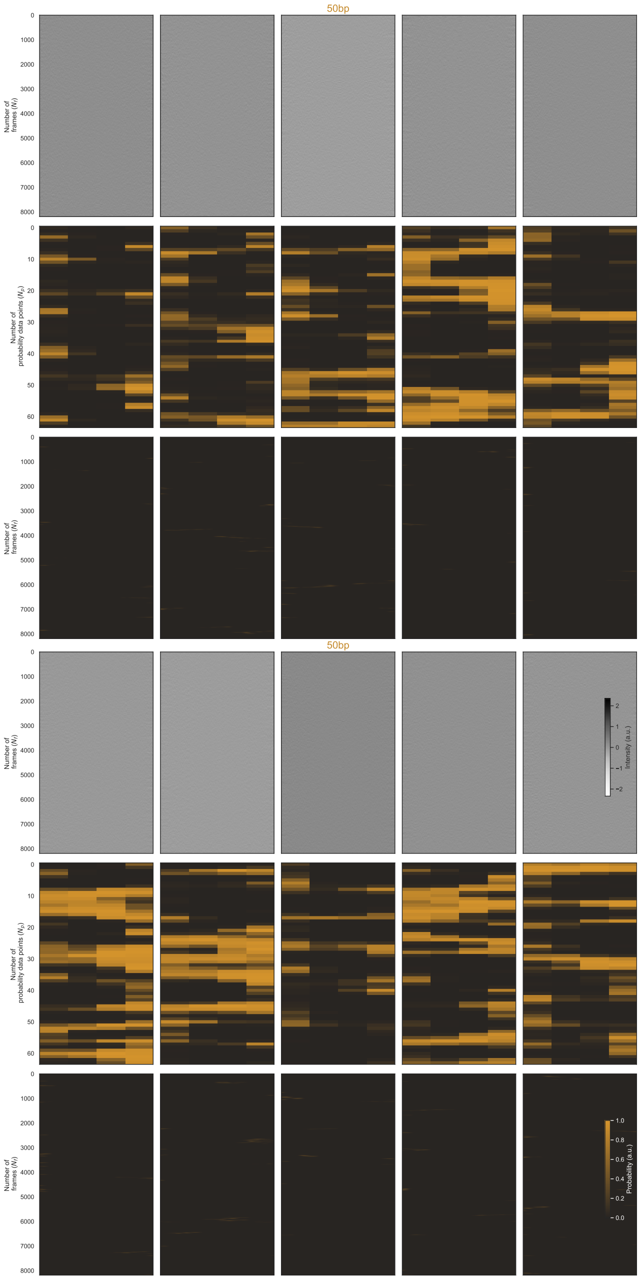

Supplementary Figure 11: Equivalent to Supplementary Figure 7, but for 50 bp double stranded DNA.

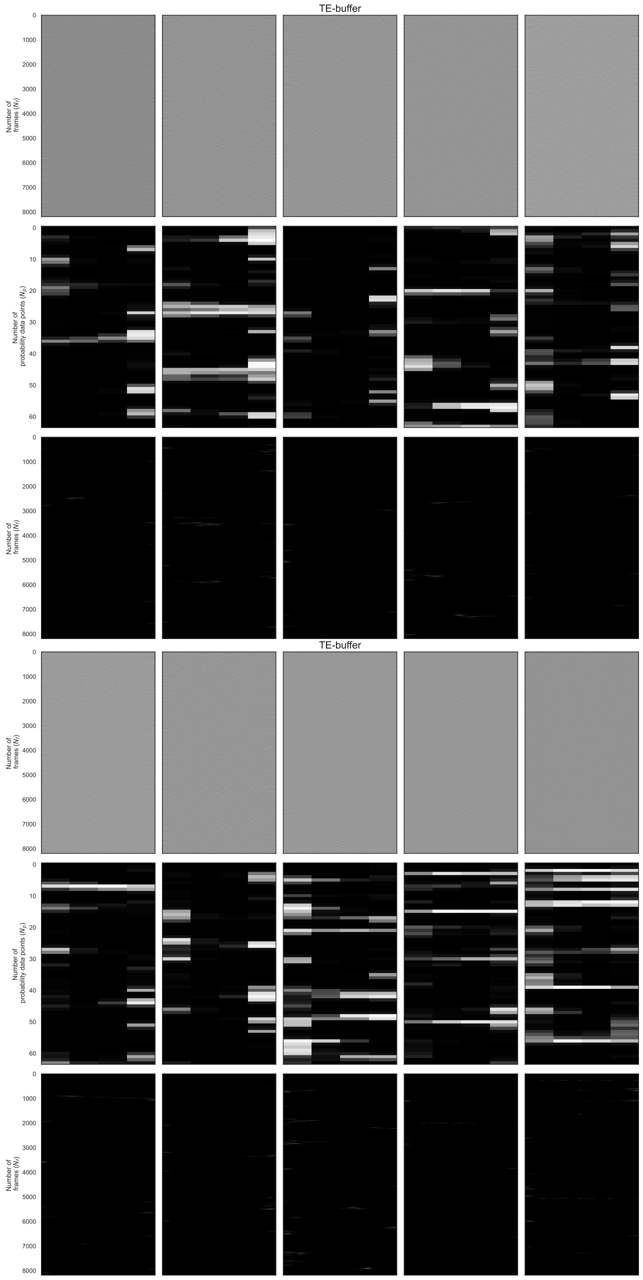

For the smallest fragment, the mean predicted MW is 26.3 ± 8.5 kDa, compared to the 30 kDa nominal value, which we consider a good agreement given the small size of the fragment. We also note that this value is very close to the mean value predicted for the empty nanochannel control with only TE-buffer, i.e. 20.5 ± 4.5 kDa.

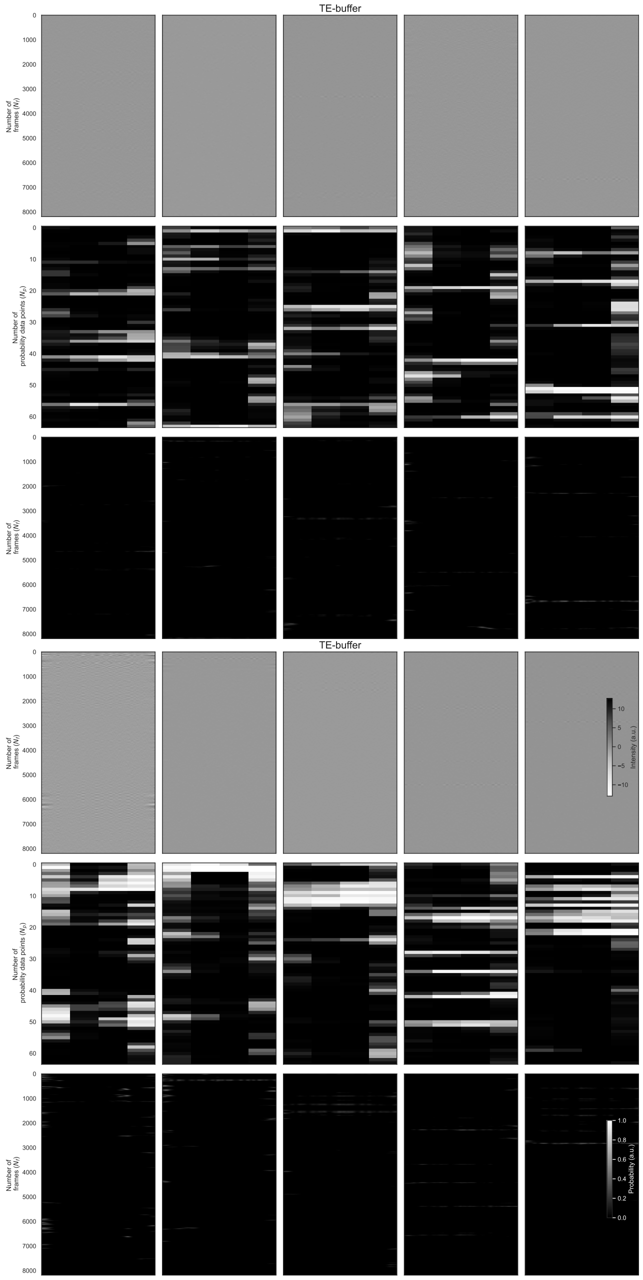

Supplementary Figure 13: Equivalent to Supplementary Figure 7, but for TE buffer.

This can be understood as that the predictions for the smallest DNA fragment (50 bp/30 kDa) indeed are approaching the noise level of the system for the given channel size, indicating that the model's detection capability is being limited by the weak scattering signal inherent to such small molecules. From the perspective of the h-ViT model, this behaviour is expected due to its probabilistic approach, which shifts the focus away from precise localization of weak signals and instead prioritizes broader property prediction based on integrated features across the kymograph. Hence, the fact that the 50 bp DNA fragment's predicted MW quite closely matches that of the TE-buffer control suggests that the signal is too weak to reliably distinguish from noise at the nanochannel and molecular size at hand. However, in local regions where the scattering signal is stronger, the h-ViT model can effectively utilize its hierarchical attention mechanisms to extract meaningful patterns from the data, resulting in more accurate predictions that align closely with the known MW. This underscores the strength of our approach to detect and characterize molecules near the LoD, while highlighting the inherent challenge in detecting extremely small biomolecules when the scattering signal is near the noise threshold.

7 Complete Insulin Measurement Data Set

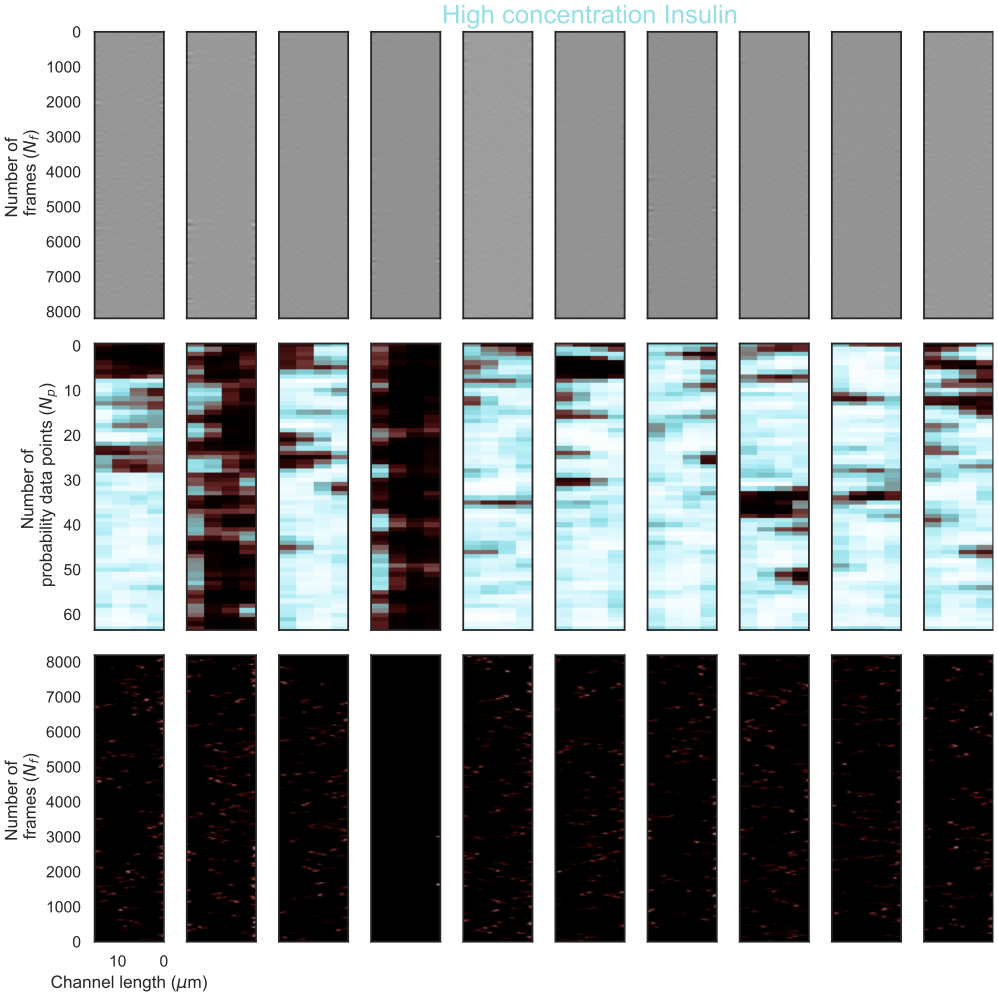

Supplementary Figure 14: **Insulin Measurements in a Nanofluidic Channel with 60 nm x 29 nm cross sectional dimension.** (**A**) Experimentally measured NSM kymographs for Insulin in PBS-buffer. (**B**) Probability maps generated by the h-ViT model using the kymograps in (A) as input. (**C**) Segmented kymographs generated by the cGAN model using the kymograps in (A) as input.

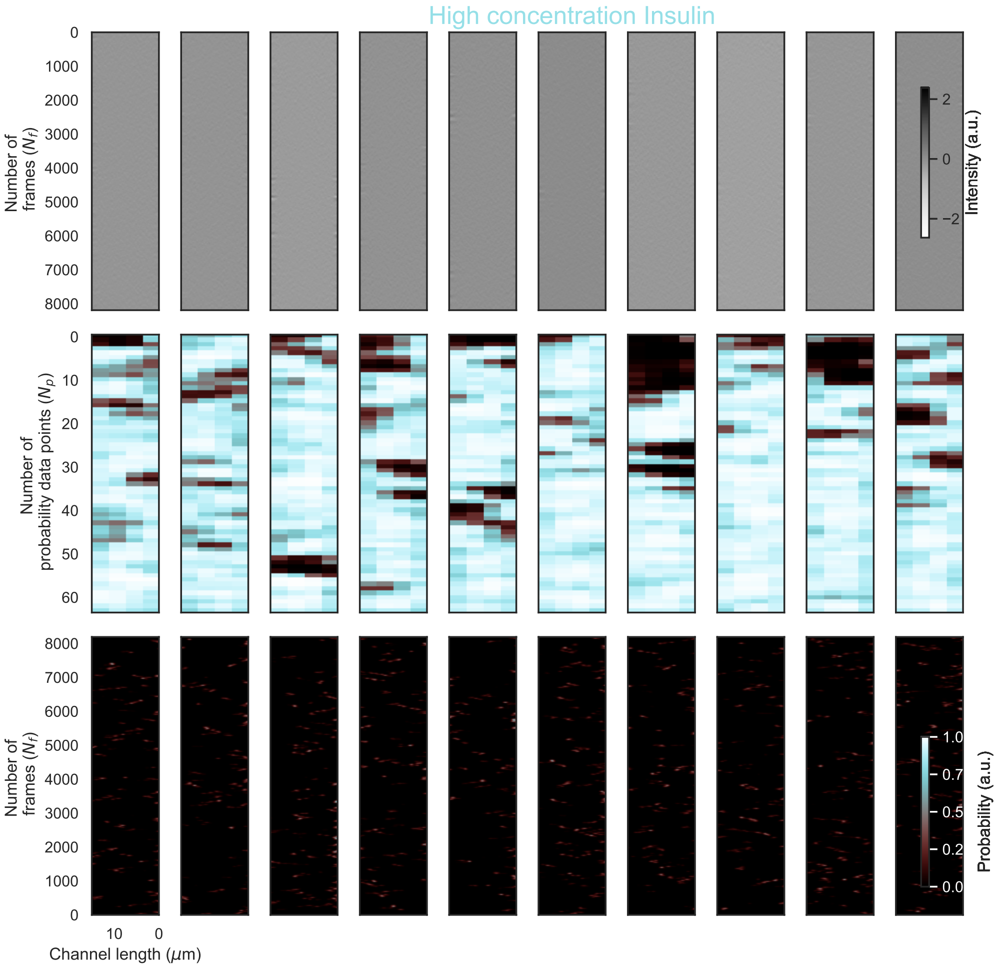

Supplementary Figure 15: Equivalent to Supplementary Figure 14.

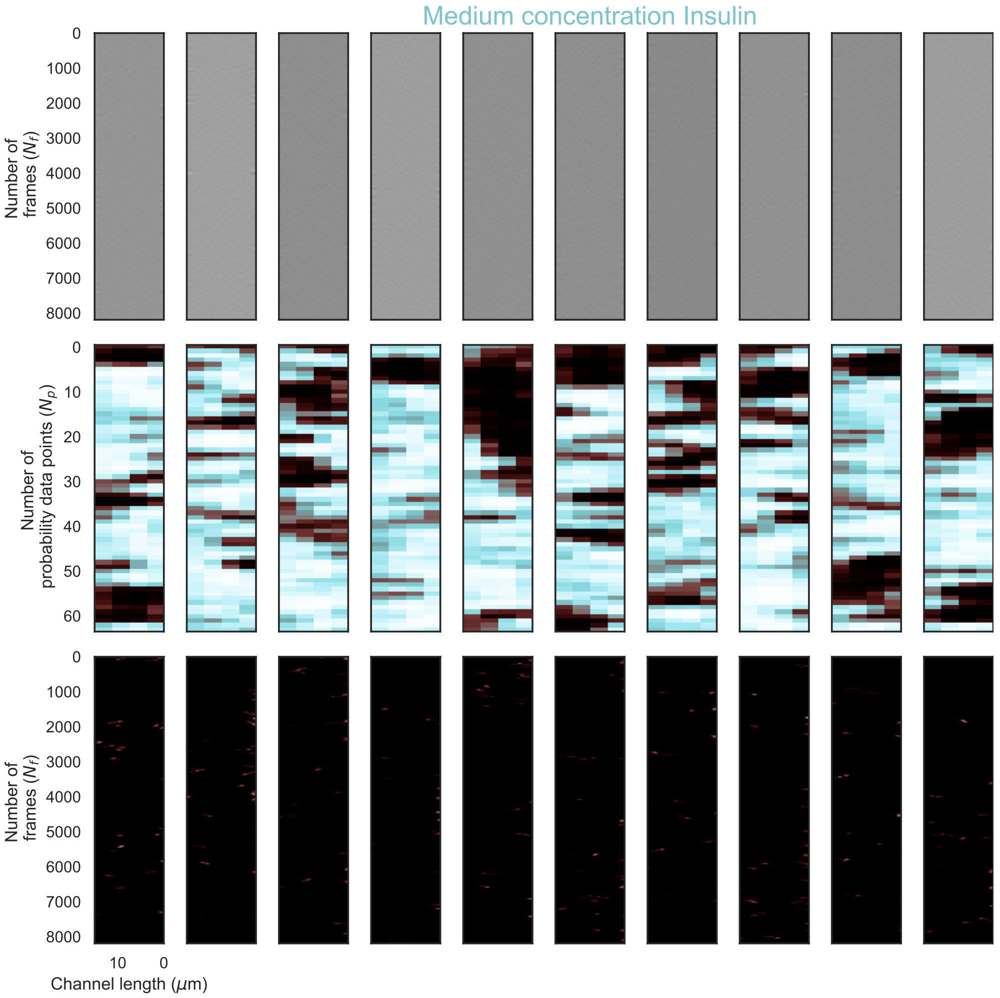

Supplementary Figure 16: Equivalent to Supplementary Figure 14, but for medium concentration Insulin.

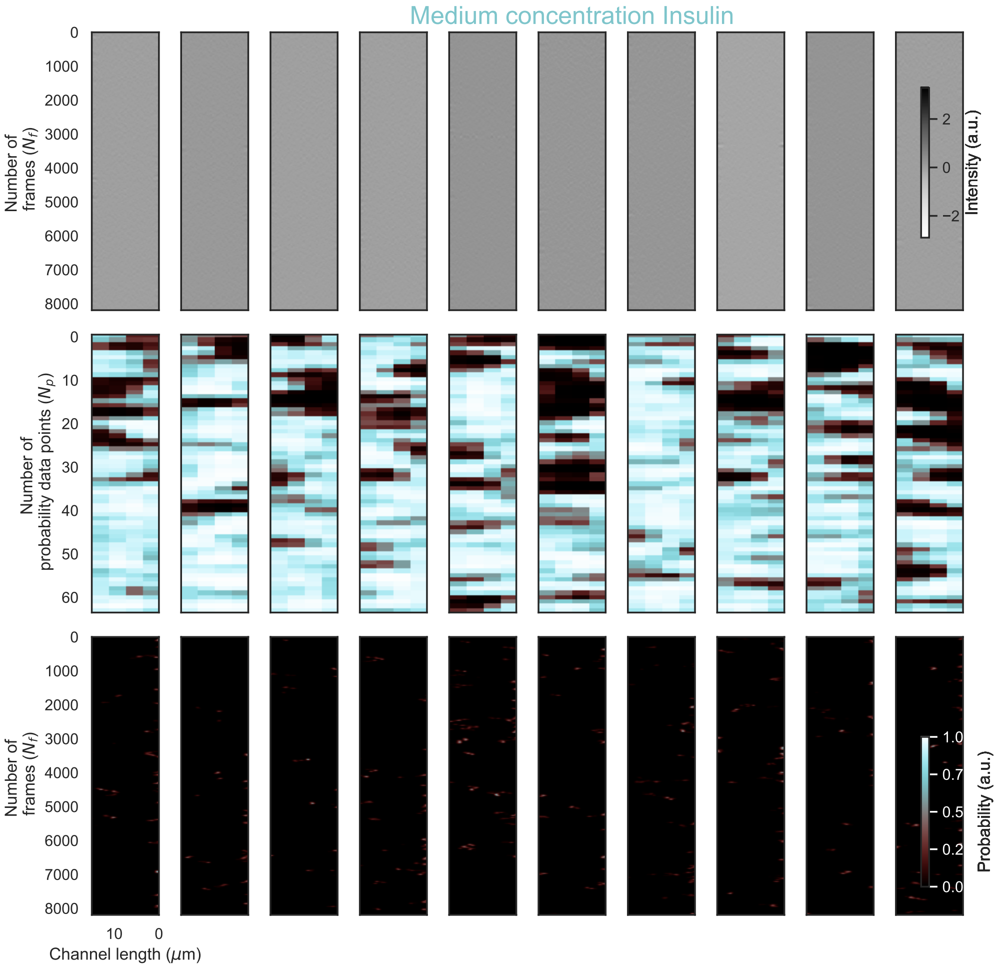

Supplementary Figure 17: Equivalent to Supplementary Figure 14, but for medium concentration Insulin.

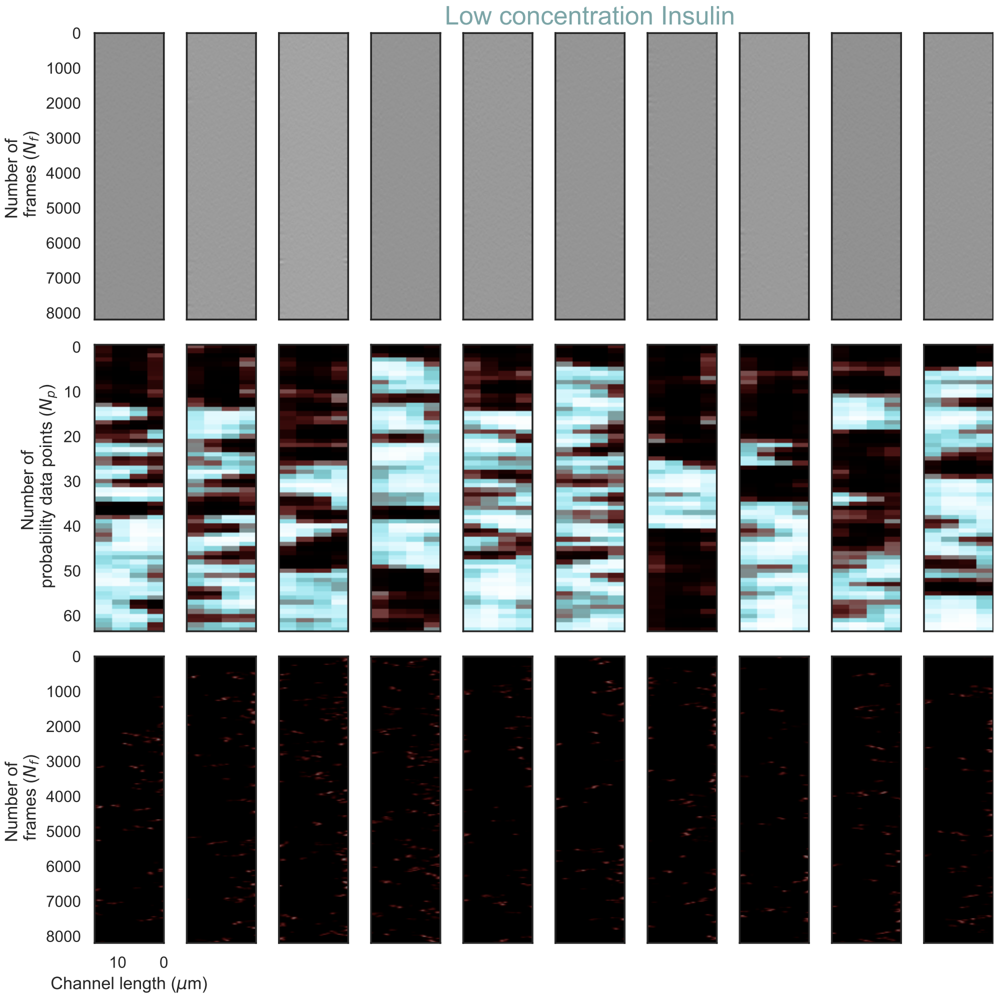

Supplementary Figure 18: Equivalent to Supplementary Figure 14, but for low concentration Insulin.

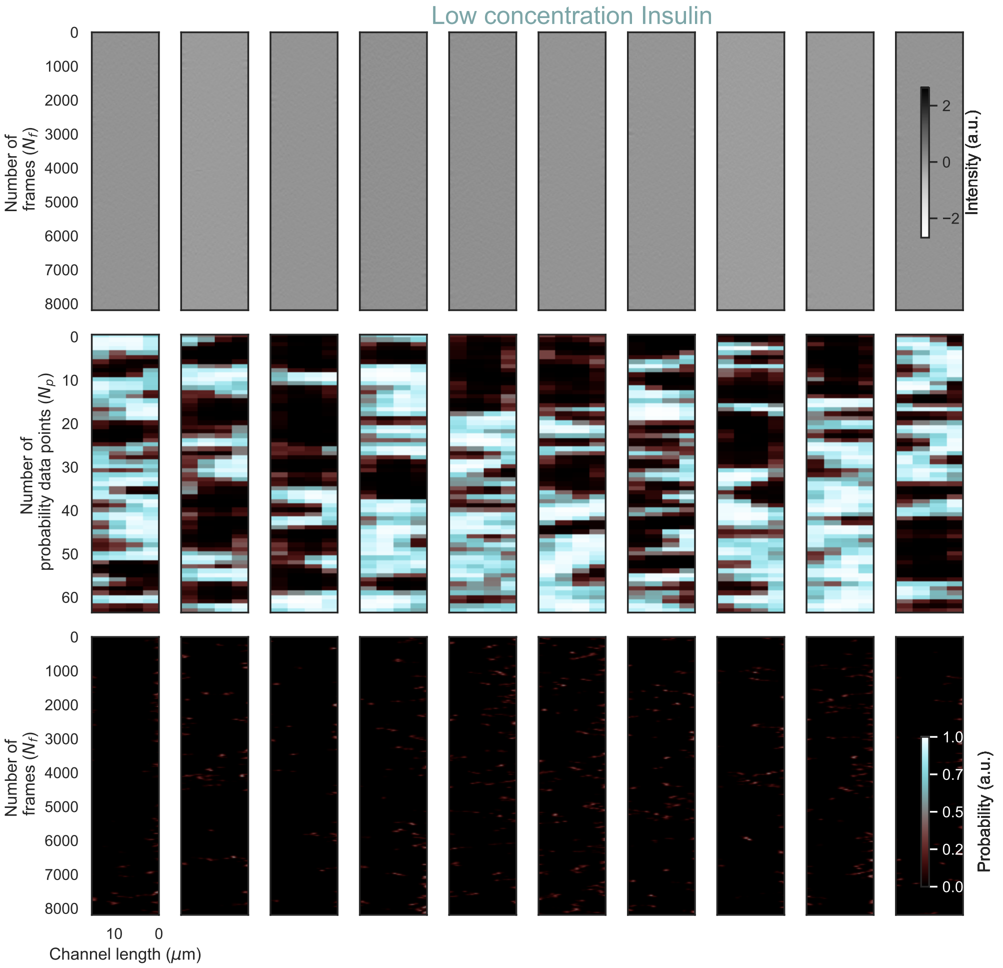

Supplementary Figure 19: Equivalent to Supplementary Figure 14, but for low concentration Insulin.

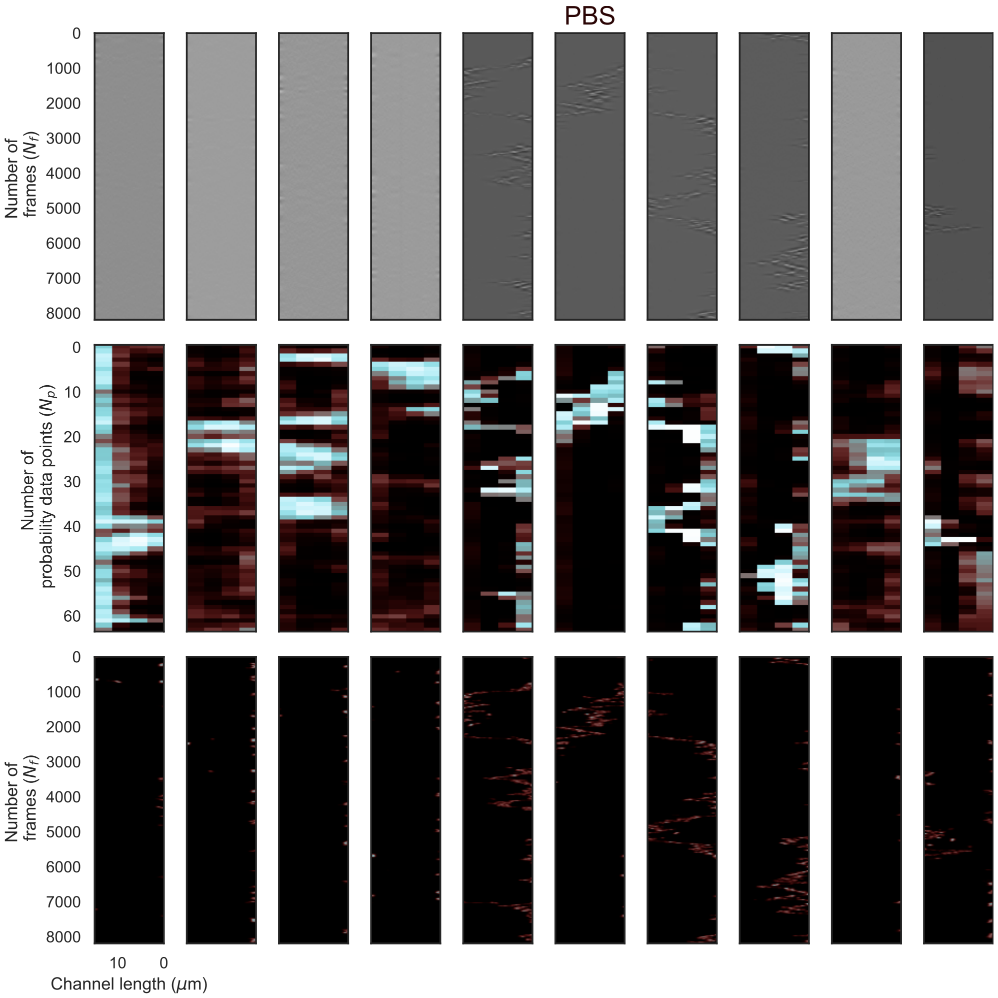

Supplementary Figure 20: Equivalent to Supplementary Figure 14, but for PBS buffer.

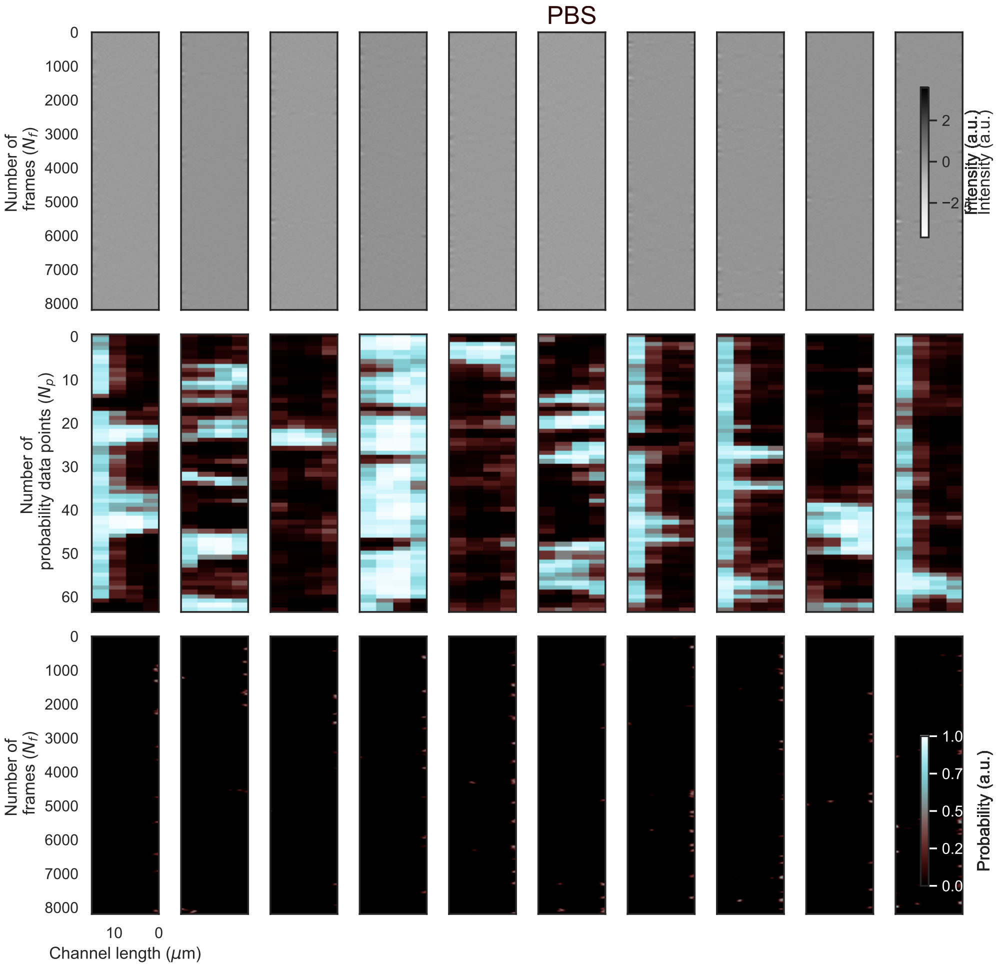

Supplementary Figure 21: Equivalent to Supplementary Figure 14, but for PBS buffer.

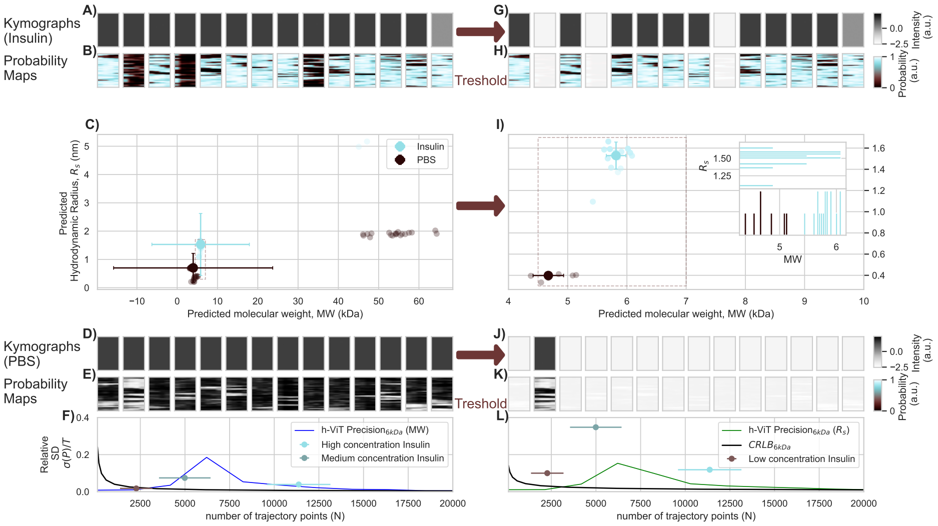

Supplementary Figure 22: **NSM Single Insulin Analysis in a Nanofluidic Channel with 29 nm x 60 nm cross-sectional dimensions**. (A) Representative kymographs for experimental Insulin measurements with NSM, serving as input to the h-ViT model. (B) Corresponding probability maps generated by the h-ViT model, indicating regions of high Insulin localization probability inside the nanochannel. (C) Predicted R_s versus MW for Insulin (turquoise) and pure PBS buffer control measurements (black), where each data point corresponds to a kymograph and the error bars correspond to the standard deviation across all measured kymographs. The transparency of each data point is correlated with the number of trajectory points within the respective kymograph. (D, E) Same as (A,B) but for nanochannels only filled with PBS buffer. (F) Relative standard deviation (σ) as function of number of trajectory points of predicted MW based on simulated trajectories for a 6 kDa molecule (blue line), experimental Insulin measurements at low, medium and high concentration (individual points) and the Cramér-Rao Lower Bound (CRLB) that illustrates the theoretical limit of prediction. (G-K) Same as (A-E) but for an applied probability threshold. (L) Same as (F) but for R_s.

Inputting the kymographs to the h-ViT model produces probability maps that highlight regions where the network has detected Insulin with high confidence **Supplementary Figure 22B**. Corresponding probability maps obtained from kymographs measured from channels only filled with PBS buffer exhibit only very low probabilities, confirming the absence of Insulin molecules in these control samples **Supplementary Figure 22E**.

Subsequently, based on these probability maps, the h-ViT model predicts the MW and R_s values for each measured kymograph for the Insulin and PBS-control samples, first

without applying any probability threshold (**Supplementary Figure 22C**). Clearly, identified molecules in the Insulin sample cluster around a common median value of MW = 5.8 ± 13.1 kDa and R_s= 1.5 ± 1.1 nm, which is equal to the nominal MW = 5.8 kDa and R_s = 1.5 nm. Nevertheless, we also notice a significant spread in the individual predicted values, as indicted by the error bars in **Supplementary Figure 22C**. This spread is the consequence of variability in the scattering signal intensity due to the inherent noise in the low SNR conditions at hand. For the pure PBS-buffer control measurements the model predicts two clusters of particles, one at lower MW and R_s than the Insulin sample and one at significantly higher values. This is the consequence of residual noise in the system being misinterpreted as ultra low-molecular-weight particles in the lower cluster when essentially no real detectable objects are in the system, and as larger objects in the higher cluster which, in principle, may correspond to real particles of a different type than Insulin. However, in particular the the data points in the higher cluster are very faint, which means they were extracted from kymographs with small N, which assigns them an inherent low reliability.

As the next step, in analogy to the DNA-ladder, we applied a threshold (see 'Post-processing' section in Methods) to the probability maps to be included in subsequent MW and R_s predictions also in this case (**Supplementary Figure 22G-K**). As expected, this thresholding step significantly improves the precision and accuracy, as evidenced by only probability maps with significantly concentrated high probabilities being included in the analysis (**Supplementary Figure 22H**) and consequently significantly narrowed predicted MW and R_s distributions (**Supplementary Figure 22I**). This is the consequence of the increased SNR in the selected probability maps, which effectively filters out low-confidence detection of molecules, and renders the identification of true insulin-related signals more consistent, and thus the MW and R_s predictions more accurate and precise. This is reflected by a much tighter distribution of the points in the scatter plot (**Supplementary Figure 22I**).

As a second aspect, we also see that the thresholding effectively eliminates the low confidence points from the PBS control data ((Supplementary Figure 22J, K) and thereby also enhances the distinction between Insulin and PBS measurements as it generates a clearer separation between the Insulin and PBS control data point clusters (**Supplementary Figure 22I**). This is the consequence of the thresholding reducing the influence of noise-driven artefacts in the PBS measurements, which are "mistaken" for small particles by the model because the model is programmed to always output predicted properties, even when the probability is very low.

As the final aspect, we discuss the absolute values of predicted median Insulin MW and R_s values after thresholding, i.e. MW = 5.85 kDa ± 0.18 kDa and R_s = 1.53 nm ± 0.13 nm (**Supplementary Figure 22I**). First, we note that the absolute values are very similar to the ones before thresholding but that the standard deviation has decreased dramatically. This advertises an impressive precision of the made predictions when a probability threshold is applied. Secondly, we note the the mean predicted Insulin MW is 0.5 kD larger than the literature value of 5.8 kDa [14]. As the main reason for this slight discrepancy we identify the level of uncertainty in accurately determining the nanochannel cross-sectional dimensions, in particular in the very small

nanochannel size regime at hand here, since such discrepancy directly affects the calibration of the iOC to MW conversion, and since its relative importance is inversely proportional to the channel cross section [2]. When it comes to the mean predicted R_s value (is 1.53 nm), we find excellent agreement with the literature value of 1.5 nm [14]. This is in line with the above argument for the slight discrepancy in MW prediction because the prediction of R_s, in contrast to the MW prediction, is insensitive to channel cross sectional dimension uncertainty, as long as the iOC is large enough to enable particle tracking, and thus derivation of D.

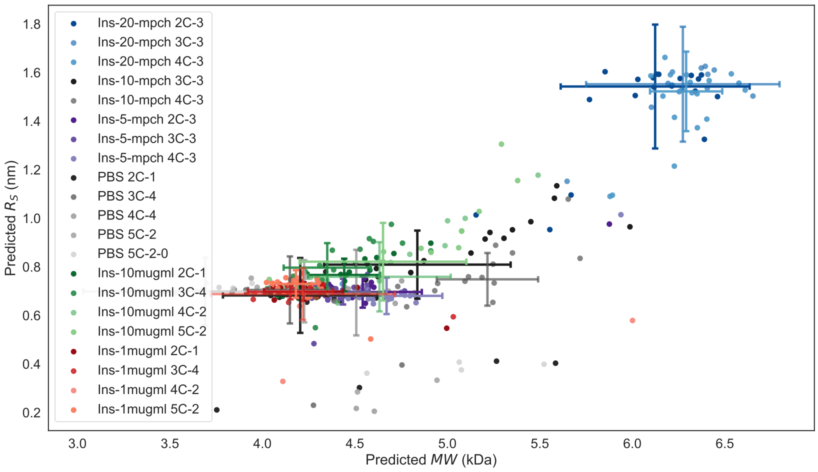

Supplementary Figure 23: Comprehensive insulin measurements across all tested concentrations and nanochannel cross-sections. We summarize NS) results for insulin acquired in multiple channels analyzed by the h-ViT. The figure establishes robustness versus concentration and channel size, and documents the few-kDa limit of detection relevant for peptide hormones.

8 Nanochannel Area Estimation

The nanochannel structures analyzed in this work are characterized by their distinctive geometric features, as shown in Supplementary Figure 24. To extract quantitative insights, we assessed the scattering cross sections through precise morphological evaluations using high-resolution scanning electron microscopy (SEM). These analyses were conducted to estimate the nanochannel area, which is crucial for determining molecular weights correlated with the optical contrast of molecules measured in our experiments.

In Supplementary Figure 24, the leftmost panel depicts the initial SEM image of a nanochannel, with a green bounding box delineating the analyzed region. The center

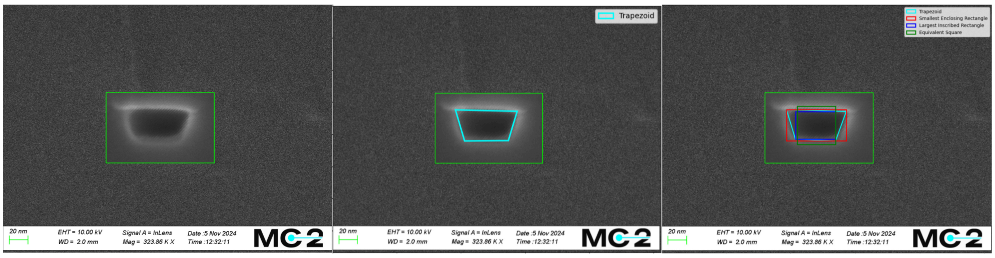

Supplementary Figure 24: Visualization of the nanochannel geometries evaluated through SEM imaging. The left panel shows the raw SEM micrograph with the region of interest outlined in green. The center panel highlights the trapezoidal fit to the nanochannel's shape, while the right panel overlays additional geometrical fits, including bounding rectangles and equivalent squares, for visual demonstration purposes. Scale bar: 20 nm.

panel demonstrates the trapezoidal approximation of the nanochannel's cross-sectional geometry, which is the sole geometric model used for area estimation and molecular weight calculations. The rightmost panel overlays additional geometric fits, including the smallest enclosing rectangle (red), the largest inscribed rectangle (blue), and the equivalent square (dark green), which are included solely for visual demonstration and are not used in any calculations.

The nanochannel area estimation directly impacts the accuracy of molecular weight determination. Variations in the trapezoidal fit introduce uncertainties in the area measurement, which propagate into the calculated molecular weights. These uncertainties, combined with intrinsic fluctuations in optical contrast, necessitate rigorous error analysis to ensure the reliability of our results. By focusing on the trapezoidal approximation and minimizing its associated uncertainties, we aim to improve the correlation between molecular weight and optical contrast, enabling more precise characterization of the molecules studied.

Following Supplementary Figure 25, the scanning electron microscopy (SEM) images depict the cross-sectional geometry of a nanofluidic channel, captured three times in identical conditions to assess its structural consistency and fabrication precision. The cross-sectional shape is approximated as a trapezoid, with measured areas of 1818, 1974, and 1880 nm^2, yielding an average of 1891 nm^2. These measurements provide critical insights into the uniformity of nanochannel fabrication, which directly impacts nanofluidic experiments.

The precise determination of the nanochannel cross-sectional area is essential for accurately estimating molecular weight in the NSM experiments. The integrated optical contrast (iOC), a key parameter in molecular weight calculations, is again inversely proportional to the nanochannel area. Consequently, variations in the channel dimensions affect iOC normalization and can introduce systematic errors in molecular weight determination. For insulin, with a molecular weight of 5.8 kDa, the accuracy of iOC measurements is particularly important due to its relatively weak scattering signal.

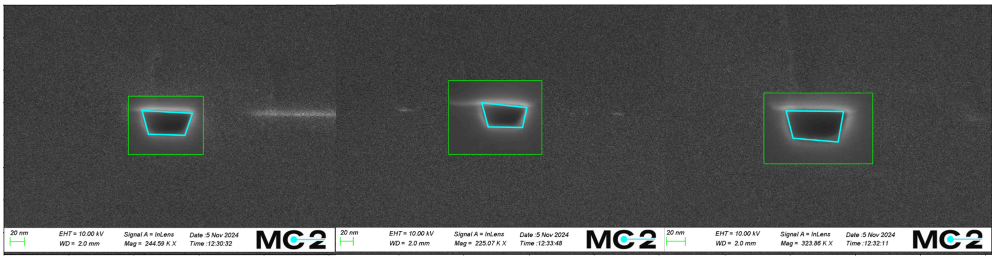

Supplementary Figure 25: Scanning electron microscopy (SEM) images of a nanofluidic channel, captured three times in identical conditions to assess geometric consistency and structural uniformity. The cross-sectional shape of the channel is approximated as a trapezoid, with estimated areas of 1828, 1964, and 1880 nm^2 based on image analysis. The mean cross-sectional area is calculated to be 1890 nm^2, which provides a crucial parameter for determining optical contrast in nanofluidic experiments. These measurements and subsequent analysis serve as a basis for evaluating nanochannel fabrication precision and its impact on single-molecule analysis within the system.

A larger-than-expected nanochannel cross-section would reduce iOC and lead to an overestimated molecular weight, while a smaller cross-section would result in an underestimated molecular weight. Given that the measured nanochannel areas exhibit a variance of approximately 8% around the mean of 1890 nm^2, this variability must be accounted for in the NSM analysis pipeline to ensure reliable molecular weight estimations.

By incorporating these nanochannel measurements into data processing and calibration procedures, systematic biases can be minimized, thereby improving the reproducibility and accuracy of molecular weight determination for small biomolecules such as insulin. Furthermore, the results highlight the necessity of precise nanochannel fabrication and post-experimental corrections to maintain the fidelity of single-molecule analysis at the lower detection limits of NSM.

8.1 Dependence on channel cross-sectional area

To explore how the model's performance depends on channel cross-sectional area, we use the same simulated dataset as above and go through a few extra steps. Firstly, we consider only predictions of simulated trajectories of length $N = 8192$ frames and (linearly) interpolate values of RME (RSD) as function of optical contrast to generate a continuous function of prediction RME (RSD) as function of optical contrast. The interpolation is achieved using SciPy [15]. Secondly, we consider nanochannel areas linearly dispersed between 50x20 and 50x120 nm, and re-calibrate the effective conversion between iOC and MW and (assuming hindered globular proteins) HR.

The cross-sectional area of the nanochannel was determined using high-resolution scanning electron microscopy (SEM) images, with the boundaries of the trapezoid identified by analyzing pixel intensity differences. To segment the nanochannel from

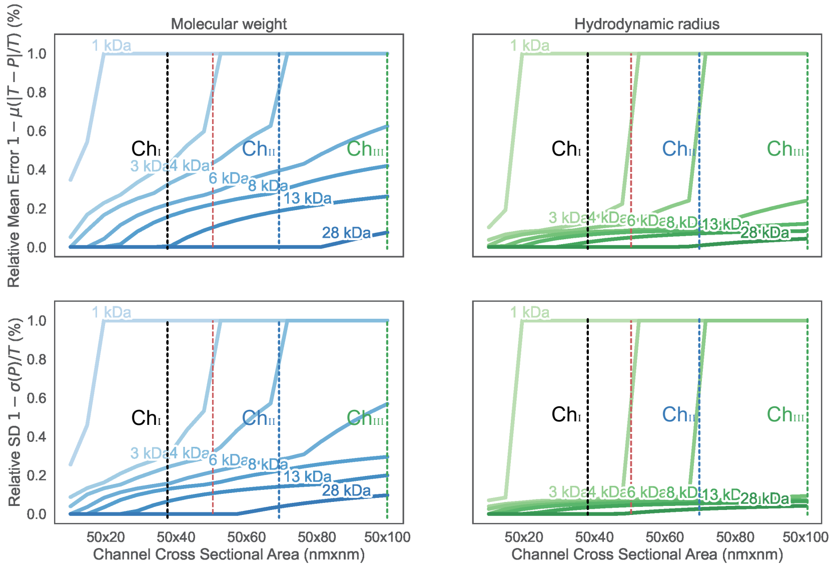

Supplementary Figure 26: Accuracy and precision of the model as a function of channel cross-sectional area. The figure illustrates the relative mean error and relative standard deviation for both molecular weight (left panels) and hydrodynamic radius (right panels) predictions across different channel sizes. The plots show how the model's performance varies with the cross-sectional area of the nanochannels, highlighting the impact of channel geometry on the estimation accuracy and precision. The dashed vertical lines in black blue and green indicate specific channel sizes (Ch_I, Ch_{II}, Ch_{III}), respectively, used in the study. The red dotted line corresponds to the size of Channel 1 in [2], used to characterize the properties of different proteins.

the surrounding material, the *Otsu thresholding algorithm* was applied to the intensity histogram of the image. This algorithm calculates an optimal intensity threshold by maximizing the variance between two pixel intensity classes: the bright boundary of the channel and the darker interior material. Specifically, Otsu's method computes the intensity threshold T that minimizes the intra-class variance, defined as:

$$\sigma_w^2(T) = q_1(T)\sigma_1^2(T) + q_2(T)\sigma_2^2(T),$$

where $q_1(T)$ and $q_2(T)$ are the probabilities of the pixel intensities being below and above T, respectively, and $\sigma_1^2(T)$ and $\sigma_2^2(T)$ are the variances of those classes. The resulting threshold T was used to binarize the SEM image, separating pixels into either the channel region or the background. The resulting binary image was overlaid onto the original SEM image for manual inspection to verify that the detected boundaries corresponded accurately to the trapezoidal geometry of the nanochannel.

To calculate the trapezoid area, the boundary points were extracted from the binary image using and the trapezoid's area was then calculated using the formula:

$$A = \frac{1}{2}(b_1 + b_2)h,$$

where b_1 and b_2 are the lengths of the parallel sides, and h is the perpendicular height between them. These dimensions were derived by converting the pixel coordinates of the vertices into physical distances using the SEM's resolution calibration factor (e.g., nanometers per pixel).

The variation in cross-sectional area, ranging from 1652 nm^2 to 1823 nm^2, arises from several interconnected factors that influence the segmentation and measurement process. While the Otsu thresholding algorithm is deterministic, its output depends on the intensity histogram of the SEM image, which is affected by noise and slight fluctuations in illumination. Noise in the image, stemming from electron beam instability or detector artifacts, can alter the intensity distribution, leading to subtle shifts in the threshold value T and, consequently, the delineation of the channel boundaries.

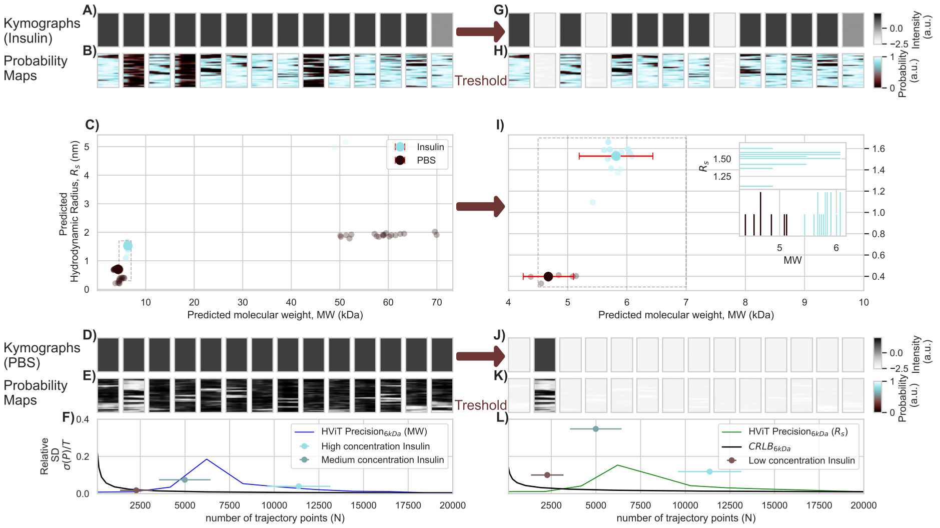

Supplementary Figure 27: Effect of nanochannel area estimation uncertainty on the inferred properties of Insulin.

The edges of the nanochannel, particularly at sloped regions, introduce ambiguity in defining the exact boundary, as pixel intensities in these regions transition gradually rather than sharply. This ambiguity results in minor variations in the placement of the

detected edges, especially when the contour-detection algorithm interpolates between pixels to trace continuous boundaries. Such interpolations, while necessary to refine the geometry, can introduce additional uncertainty in the measured dimensions of b_1, b_2, and h.

Moreover, the calibration factor used to convert pixel dimensions to physical distances introduces a systematic source of variation. Even minor inaccuracies in the SEM's magnification settings or calibration standards can propagate through the area calculations, compounding the observed range. Manual inspection of the binary image, used to validate the detected boundaries, also contributes to variability, as human judgment in confirming or adjusting edges can be influenced by noise and resolution limits.

Taken together, these factors-histogram sensitivity, edge ambiguity, resolution limits, and calibration variability-interact to produce the range of 1652 nm^2 to 1823 nm^2. This range reflects the inherent uncertainty of the measurement process, providing a robust estimate of the nanochannel's cross-sectional area. This variability in area estimation directly affects the predicted molecular weight of insulin, as reflected in the red error bars in Supplementary Figure 27, underscoring the impact of this measurement uncertainty in the estiamation of molecular properties in this regime.

9 Quantitative Comparison of Simulated and Experimental Kymographs

In this section we provide a rigorous statistical analysis of the similarity ("domain gap") between simulated and experimental diffusion kymographs used throughout this work. Twenty simulated and twenty experimental kymographs were randomly selected (without replacement) from their respective corpora to form balanced comparison subsets. Each kymograph is treated as a two-dimensional real-valued intensity field $I(x, t)$ after standard pre-processing (background subtraction, temporal normalization) identical to that applied prior to model training, implemented in Python using NumPy [16] and SciPy [17].

For every kymograph K we compute a vector of handcrafted features capturing first-order photometric statistics, distributional shape, and local spatial structure. Let $S_K = \{I_{ij}\}_{i=1,\ldots,H;j=1,\ldots,W}$ denote all pixel intensities after flattening. We define:

$$\mu_K = \frac{1}{HW}\sum_{i,j} I_{ij}, \qquad \sigma_K = \sqrt{\frac{1}{HW}\sum_{i,j}(I_{ij} - \mu_K)^2}, \tag{7}$$

$$I_{\min,K} = \min_{i,j} I_{ij}, \qquad I_{\max,K} = \max_{i,j} I_{ij}. \tag{8}$$

Further, for a percentile set $P = \{1, 5, 25, 50, 75, 95, 99\}$ we compute $q_{p,K}$ = empirical percentile at level p of S_K. To incorporate local structural variation we approximate

the spatial gradient magnitude using forward differences:

$$G_{ij} = \sqrt{(\nabla_x I_{ij})^2 + (\nabla_y I_{ij})^2}, \qquad \bar{G}_K = \frac{1}{HW}\sum_{i,j} G_{ij}, \qquad s_{G,K} = \sqrt{\frac{1}{HW}\sum_{i,j}(G_{ij} - \bar{G}_K)^2}. \tag{9}$$

The final feature vector is

$$\mathbf{f}_K = (\mu_K,\, \sigma_K,\, I_{\min,K},\, I_{\max,K},\, q_{1,K}, q_{5,K}, q_{25,K}, q_{50,K}, q_{75,K}, q_{95,K}, q_{99,K}, \bar{G}_K, s_{G,K})^\top \in \mathbb{R}^{13}. \tag{10}$$

These features (dimension reported in Table 3) are chosen to align with the photometric determinants of contrast and detectability, and remain computationally light, enabling rapid resampling analyses.

To compare global photometric statistics we construct normalized histograms. Let $[a, b]$ be the joint intensity range over all 40 kymographs. We define B equally spaced bin edges $a = e_0 < e_1 < \cdots < e_B = b$ with $B = 128$. For domain $D \in \{\text{Sim}, \text{Exp}\}$ containing kymographs $K \in D$, the (per-kymograph) density estimate for bin k is

$$h_K(k) = \frac{1}{N_K}\sum_{i,j} \mathbf{1}[e_k \le I_{ij} < e_{k+1}], \qquad N_K = HW. \tag{11}$$

The domain-mean histogram is $\bar{h}_D(k) = |D|^{-1}\sum_{K\in D} h_K(k)$, which satisfies $\sum_k \bar{h}_D(k) = 1$. All divergence metrics below are computed on $\bar{h}_{\text{Sim}}$ and $\bar{h}_{\text{Exp}}$. We apply an additive smoothing $\epsilon = 10^{-9}$ when required for numerical stability.

9.1 Histogram-Based Divergences and Distances

Given two discrete distributions $p = (p_k)$ and $q = (q_k)$ we report classical L_1 and L_2 distances, Jensen–Shannon divergence [18], Kullback–Leibler divergence [19], the first Wasserstein (earth mover's) distance [20], and the Kolmogorov–Smirnov statistic [21, 22]:

$$extL_1(p, q) = \sum_k |p_k - q_k|, \qquad \mathrm{L}_2(p, q) = \left(\sum_k (p_k - q_k)^2\right)^{1/2}. \tag{12}$$

The (symmetric) Jensen-Shannon divergence (base e) is

$$\mathrm{JS}(p, q) = \tfrac{1}{2}D_{\mathrm{KL}}(p \,\|\, m) + \tfrac{1}{2}D_{\mathrm{KL}}(q \,\|\, m), \qquad m = \tfrac{1}{2}(p + q), \tag{13}$$

with Kullback-Leibler divergence $D_{\mathrm{KL}}(p \,\|\, q) = \sum_k p_k \log \frac{p_k}{q_k}$. The first Wasserstein distance for 1D intensities treats each bin center $c_k = \frac{1}{2}(e_k + e_{k+1})$ as support point and is computed via the cumulative distribution difference. The Kolmogorov-Smirnov

statistic is applied to random subsamples (up to 2×10^5 pixels per domain) drawn without replacement from pooled pixel intensities to approximate the difference between empirical cumulative distribution functions.

9.2 Multivariate Feature Space Metrics

Let feature matrices $F_{\text{Sim}} \in R^{n_s \times d}$ and $F_{\text{Exp}} \in R^{n_e \times d}$ collect row-wise feature vectors with $d = 13$. We define sample means $\boldsymbol{\mu}_s, \boldsymbol{\mu}_e$ and unbiased covariances C_s, C_e. The Fréchet-like distance (FID-like) employed (analogue of Inception-based FID [23] on handcrafted features) is

$$\text{FID}_{\text{hand}} = \|\boldsymbol{\mu}_s - \boldsymbol{\mu}_e\|_2^2 + \text{Tr}\left(C_s + C_e - 2(C_s C_e)^{1/2}\right), \tag{14}$$

where $(C_s C_e)^{1/2}$ is the principal matrix square root, small imaginary parts due to numerical rounding are discarded. Diagonal jitter $10^{-6} I_d$ is added prior to the square root for stability. Because features are low-dimensional handcrafted statistics (not deep embeddings), absolute values of FID_{hand} are only interpretable comparatively. The (unbiased) squared Maximum Mean Discrepancy (MMD) [24] with multi-scale radial basis function (RBF) kernels uses a bandwidth set $\Gamma = \{0.01, 0.05, 0.1, 0.5, 1, 2, 5\}$ applied to pairwise squared Euclidean distances:

$$\text{MMD}^2(F_s, F_e) = \frac{1}{m(m-1)} \sum_{i \neq j} k(\mathbf{f}_i, \mathbf{f}_j) + \frac{1}{n(n-1)} \sum_{i \neq j} k(\mathbf{g}_i, \mathbf{g}_j) - \frac{2}{mn} \sum_{i,j} k(\mathbf{f}_i, \mathbf{g}_j), \tag{15}$$

with $k(u, v) = \frac{1}{|\Gamma|} \sum_{\gamma \in \Gamma} \exp(-\gamma \|u - v\|_2^2)$, and $m = n = 20$ here. We report the averaged MMD^2 (denoted MMD in Table 3).

All computations were performed in Python (NumPy/SciPy, custom functions) with deterministic random number generation (seed 42) for pixel subsampling. Histogram bin count (128) was selected empirically to balance resolution and variance given $\mathcal{O}(10^6)$ total pixels per subset. Gradient magnitudes were computed with first-order finite differences (`numpy.gradient`) and cast to 32-bit floating point prior to aggregation. All scalar divergences are printed with full precision but rounded for tabular presentation.

Supplementary Figure 28A shows near-perfect overlap between domain-mean intensity distributions, consistent with the very small JS divergence (0.00374) and moderate Wasserstein distance (0.0645). Supplementary Figure 28B reveals partial but not complete overlap in the (mean, standard deviation) plane, indicating modest elevation in intensity variance for a subset of experimental kymographs. The L_1 and L_2 histogram distances indicate only minor redistribution of probability mass across bins. The KS statistic (0.038) attains an extreme p-value owing to the very high pixel sample size and should not be over-interpreted as practical divergence. The FID-like value (53.35) and multi-scale MMD (0.0966) quantify a moderate shift driven principally by higher-order and gradient-derived components of $\mathbf{f}_K$. Overall, photometric alignment (Panel A) is excellent, while higher-order structural variability (Panel B and multivariate metrics) leaves limited headroom for possible future refinement (e.g. injection

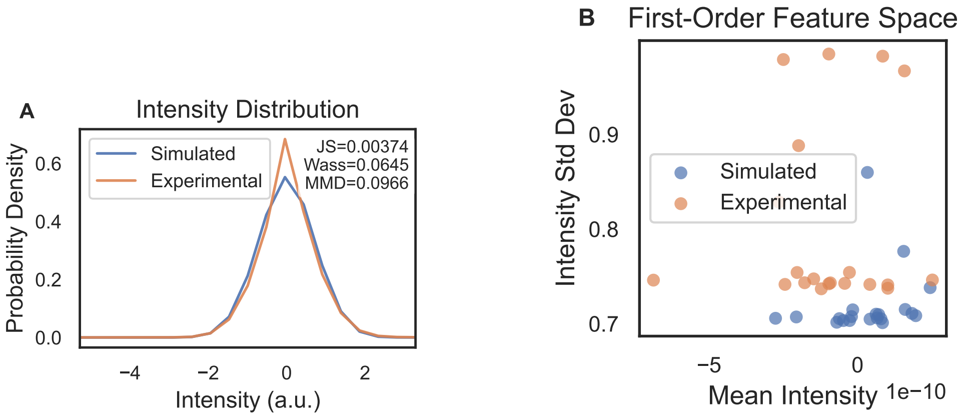

Supplementary Figure 28: **Quantitative comparison of simulated and experimental kymographs.** A: domain-mean marginal intensity distributions (trimmed to 99.8% mass) show close photometric alignment with low divergence values. Only the central intensity region is displayed to improve readability. Truncation indices are chosen as the smallest and largest bin centers whose cumulative average histogram mass reaches 0.001 and 0.999 respectively. This retains 99.8% of probability mass while excluding extreme tails dominated by sparse noise. B: first-order feature space (mean vs standard deviation) reveals modest variance shift between domains, underpinning multivariate distance metrics.

Supplementary Table 3: Domain shift metrics for simulated versus experimental kymographs (20 per domain). Lower values generally indicate greater similarity. JS: Jensen-Shannon divergence; $\text{KL}_{P\to Q}$: KL divergence from simulated to experimental; Wass: 1-Wasserstein distance; FID-like: Fréchet-style distance on handcrafted features; MMD: multi-scale RBF Maximum Mean Discrepancy.

Metric	Value
Number of kymographs (Sim / Exp)	20 / 20
JS divergence	0.00374
$\text{KL}_{Sim\to Exp}$	0.01397
$\text{KL}_{Exp\to Sim}$	0.01854
Histogram L_1 distance	0.2928
Histogram L_2 distance	0.1478
Wasserstein distance	0.06445
KS statistic	0.0380
FID-like (handcrafted)	53.35
RBF MMD	0.0966
Feature dimensionality	13

of empirically calibrated noise textures). These findings justify the use of the simulated corpus for training without introducing substantial bias in intensity-dependent downstream quantities.

Table 3 compiles all scalar metrics alongside feature dimensionality to facilitate reproducibility. All metrics can be re-generated deterministically by re-running the analysis function with identical seed and sampling parameters.

The analysis demonstrates strong photometric parity and only modest higher-order structural shift, supporting the fidelity of the simulation pipeline for model development.

10 Cramér-Rao Lower Bound (CRLB)

The Cramér-Rao Lower Bound (CRLB) provides a theoretical lower bound on the variance of any unbiased estimator of a parameter, indicating the best precision achievable under a given statistical model [25, 26]. It is a fundamental concept in estimation theory and is widely used in statistical signal processing [27]. The CRLB is directly tied to the Fisher information, denoted as $I(\theta)$, which measures the amount of information that an observable random variable conveys about an unknown parameter θ. The mathematical expression for the CRLB is given by:

$$\mathrm{Var}(\hat{\theta}) \geq \frac{1}{I(\theta)}$$

where $\mathrm{Var}(\hat{\theta})$ represents the variance of the estimator $\hat{\theta}$. This inequality suggests that the variance of any unbiased estimator cannot be smaller than the inverse of the Fisher information.

It serves as a benchmark for assessing the efficiency of estimators. An estimator that achieves this lower bound is considered efficient, as it has the smallest possible variance among all unbiased estimators for that parameter. The amount of data influences the Fisher information-generally, an increase in data leads to higher Fisher information, which implies a tighter bound and hence, a reduced potential estimation error.

10.0.1 Define the Statistical Model

We begin by specifying the probability density function (pdf) or probability mass function (pmf) of our data, including the scale parameter σ that we aim to estimate. The choice of model depends on the nature of our data (e.g., normal distribution, exponential distribution).

Example: Consider a normal distribution with mean zero and unknown scale (standard deviation) σ:

$$f(x;\sigma) = \frac{1}{\sqrt{2\pi}\sigma} \exp\left(-\frac{x^2}{2\sigma^2}\right)$$

This model is appropriate for many natural phenomena due to the Central Limit Theorem [28].

10.0.2 Find the Likelihood Function

For a sample of independent and identically distributed observations $X = (X_1, X_2, \ldots, X_n)$, we construct the likelihood function by taking the product of individual pdfs:

$$L(\sigma; X) = \prod_{i=1}^{n} f(x_i; \sigma)$$

Example:

$$L(\sigma; X) = \left(\frac{1}{\sqrt{2\pi}\sigma} \right)^n \exp\left(-\frac{\sum_{i=1}^{n} x_i^2}{2\sigma^2} \right)$$

10.0.3 Compute the Log-Likelihood Function

Next, we take the natural logarithm of the likelihood function to simplify differentiation:

$$\ell(\sigma; X) = \ln L(\sigma; X)$$

Example:

$$\ell(\sigma; X) = -n \ln \sigma - \frac{\sum_{i=1}^{n} x_i^2}{2\sigma^2} + \text{constant terms}$$

10.0.4 Calculate the First Derivative (Score Function)

We differentiate the log-likelihood function with respect to σ to obtain the score function:

$$\frac{\partial \ell(\sigma; X)}{\partial \sigma} = -\frac{n}{\sigma} + \frac{\sum_{i=1}^{n} x_i^2}{\sigma^3}$$

10.0.5 Compute the Second Derivative

We now differentiate the score function with respect to σ to get the observed information:

$$\frac{\partial^2 \ell(\sigma; X)}{\partial \sigma^2} = \frac{n}{\sigma^2} - \frac{3 \sum_{i=1}^{n} x_i^2}{\sigma^4}$$

10.0.6 Calculate the Fisher Information $I(\sigma)$

The Fisher Information quantifies the amount of information that our observable data carries about the unknown parameter σ:

$$I(\sigma) = -\mathrm{E}\left[\frac{\partial^2 \ell(\sigma; X)}{\partial \sigma^2}\right]$$

Given that $X_i \sim N(0, \sigma^2)$ and $\mathrm{E}[X_i^2] = \sigma^2$, we have:

$$I(\sigma) = -\left(\frac{n}{\sigma^2} - \frac{3n\sigma^2}{\sigma^4}\right) = \frac{2n}{\sigma^2}$$

This result is consistent with standard statistical texts [29].

10.0.7 Compute the Cramér-Rao Lower Bound

Finally, the CRLB provides the minimum variance bound for any unbiased estimator $\hat{\sigma}$ of σ:

$$\mathrm{Var}(\hat{\sigma}) \geq \frac{1}{I(\sigma)} = \frac{\sigma^2}{2n}$$

This implies that no unbiased estimator of σ can have a variance lower than $\frac{\sigma^2}{2n}$, as established in estimation theory [27].

10.1 Cramér-Rao Lower Bound for NSM

In NSM, the intensity of scattered light from biomolecules diffusing in a nanofluidic channel provides insights into molecular weight and hydrodynamic radius. For NSM, the observed intensity I_t of the scattered light from a biomolecule within a nanochannel can be modeled as:

$$I_t = cI_0L|\alpha_t|^2\frac{k^3}{4}$$

where:

- $\alpha_t = \alpha_c + \dfrac{\alpha_m}{L^2}$ represents the total polarizability, composed of the polarizability of the nanochannel α_c and the biomolecule α_m.
- α_m, α_c: Polarizabilities of the biomolecule and the nanochannel.
- I_0: Incident light intensity.
- k: Wavenumber of the light.
- L: Length of the illuminated part of the nanochannel.
- c: Collection efficiency.

10.1.1 Assumptions for the Likelihood Function

Assume the measured intensity I_t is subject to Gaussian noise. Thus, the probability density function of observing I_t given the parameters α_m is modeled as:

$$f(I_t; \alpha_m) = \frac{1}{\sqrt{2\pi}\sigma} \exp\left(-\frac{(I_t - \mu(\alpha_m))^2}{2\sigma^2}\right)$$

where:

$$\mu(\alpha_m) = cI_0L\left(\alpha_c + \frac{\alpha_m}{L^2}\right)^2 \frac{k^3}{4}$$

and σ is the standard deviation of the measurement noise.

The log-likelihood function $\log f(I_t; \alpha_m)$ is:

$$\log f(I_t; \alpha_m) = -\frac{1}{2}\log(2\pi\sigma^2) - \frac{(I_t - \mu(\alpha_m))^2}{2\sigma^2}$$

10.1.2 Derivation of the Fisher Information

The Fisher Information $I(\alpha_m)$ is defined as the negative expected value of the second derivative of the log-likelihood with respect to the parameter α_m. In the Gaussian noise model with known variance, the Fisher Information simplifies to:

$$I(\alpha_m) = \frac{1}{\sigma^2}\left(\frac{\partial\mu(\alpha_m)}{\partial\alpha_m}\right)^2$$

Compute the first derivative of $\mu(\alpha_m)$ with respect to α_m:

$$\frac{\partial\mu(\alpha_m)}{\partial\alpha_m} = cI_0L \cdot 2\left(\alpha_c + \frac{\alpha_m}{L^2}\right) \cdot \left(\frac{1}{L^2}\right) \cdot \frac{k^3}{4}$$

Simplify:

$$\frac{\partial\mu(\alpha_m)}{\partial\alpha_m} = \frac{cI_0k^3}{2L}\left(\alpha_c + \frac{\alpha_m}{L^2}\right)$$

Note that $\alpha_t = \alpha_c + \frac{\alpha_m}{L^2}$, so:

$$\frac{\partial\mu(\alpha_m)}{\partial\alpha_m} = \frac{cI_0k^3}{2L}\alpha_t$$

Therefore, the Fisher Information is:

$$I(\alpha_m) = \frac{1}{\sigma^2}\left(\frac{cI_0k^3}{2L}\alpha_t\right)^2$$

10.1.3 Cramér-Rao Lower Bound (CRLB)

The CRLB provides a lower bound on the variance of any unbiased estimator of α_m:

$$\mathrm{Var}(\hat{\alpha}_m) \geq \frac{1}{I(\alpha_m)} = \frac{\sigma^2}{\left(\frac{cI_0k^3}{2L}\alpha_t\right)^2}$$

Simplify:

$$\mathrm{Var}(\hat{\alpha}_m) \geq \sigma^2 \left(\frac{2L}{cI_0k^3\alpha_t}\right)^2$$

This expression shows that the variance of the estimator depends on the true value of α_t, which includes the parameter α_m we aim to estimate.

10.1.4 Model for Multiple Measurements

For N independent measurements, the total Fisher Information is:

$$I_{\text{total}}(\alpha_m) = N \times I(\alpha_m)$$

Thus, the CRLB for estimating α_m from N measurements is:

$$\mathrm{Var}(\hat{\alpha}_m) \geq \frac{1}{I_{\text{total}}(\alpha_m)} = \frac{\sigma^2}{N}\left(\frac{2L}{cI_0k^3\alpha_t}\right)^2$$

10.1.5 CRLB for Diffusivity in NSM

The relationship between the diffusivity D and the hydrodynamic radius R_s is given by the Stokes-Einstein equation:

$$D = \frac{k_BT}{6\pi\eta R_\text{s}}$$

Considering the displacement x of a diffusing particle over time t, the probability density function is:

$$f(x; D) = \frac{1}{\sqrt{4\pi Dt}} \exp\left(-\frac{x^2}{4Dt}\right)$$

The log-likelihood function is:

$$\log f(x; D) = -\frac{1}{2}\log(4\pi Dt) - \frac{x^2}{4Dt}$$

Compute the first derivative with respect to D:

$$\frac{\partial}{\partial D}\log f = -\frac{1}{2D} + \frac{x^2}{4D^2t}$$

Compute the second derivative:

$$\frac{\partial^2}{\partial D^2} \log f = \frac{1}{2D^2} - \frac{x^2}{2D^3 t}$$

Calculate the Fisher Information by taking the negative expected value of the second derivative. Since $E[x^2] = 2Dt$, we have:

$$I(D) = -E\left[\frac{\partial^2}{\partial D^2} \log f\right] = \frac{1}{2D^2}$$

Thus, the CRLB for a single measurement is:

$$\text{Var}(\hat{D}) \geq \frac{1}{I(D)} = 2D^2$$

For N independent measurements:

$$I_{\text{total}}(D) = N \times I(D) = \frac{N}{2D^2}$$

So the CRLB becomes:

$$\text{Var}(\hat{D}) \geq \frac{1}{I_{\text{total}}(D)} = \frac{2D^2}{N}$$

10.1.6 Final CRLB Expressions

$$\boxed{\text{Var}(\hat{\alpha}_m) \geq \frac{\sigma^2}{N}\left(\frac{2L}{cI_0 k^3 \alpha_t}\right)^2}$$

$$\boxed{\text{Var}(\hat{D}) \geq \frac{2D^2}{N}}$$

10.2 Including Localization Error in CRLB

So far, we have assumed a theoretically optimal but practically impossible localization error of 0. If we include the theoretical limits of localization error,for n independent displacement measurements over a total observation time $T = n\Delta t$, the CRLB is derived as follows.

Let σ_L be the standard deviation of the localization error. The measured position $x_m(t)$ at time t is:

$$x_m(t) = x(t) + \epsilon$$

where $x(t)$ is the true position, and ϵ is a zero-mean Gaussian random variable with variance σ_L^2.

The observed displacement Δx_m between two time points separated by Δt is:

$$\Delta x_m = x_m(t + \Delta t) - x_m(t) = \Delta x + \epsilon_2 - \epsilon_1$$

where $\Delta x = x(t + \Delta t) - x(t)$ is the true displacement due to diffusion.

The variance of the observed displacement Δx_m is:

$$\mathrm{Var}(\Delta x_m) = 2D\Delta t + 2\sigma_L^2$$

10.2.1 Likelihood Function and Fisher Information

Assuming that the observed displacements Δx_m are independent and normally distributed, the likelihood function for observing a displacement Δx_m given diffusivity D is:

$$f(\Delta x_m; D) = \frac{1}{\sqrt{4\pi(D\Delta t + \sigma_L^2)}} \exp\left(-\frac{(\Delta x_m)^2}{4(D\Delta t + \sigma_L^2)}\right)$$

The log-likelihood function is:

$$\log f(\Delta x_m; D) = -\frac{1}{2}\log\left(4\pi(D\Delta t + \sigma_L^2)\right) - \frac{(\Delta x_m)^2}{4(D\Delta t + \sigma_L^2)}$$

The first derivative with respect to D is:

$$\frac{\partial}{\partial D}\log f = -\frac{\Delta t}{2(D\Delta t + \sigma_L^2)} + \frac{(\Delta x_m)^2 \Delta t}{4(D\Delta t + \sigma_L^2)^2}$$

The second derivative is:

$$\frac{\partial^2}{\partial D^2}\log f = \frac{\Delta t^2}{2(D\Delta t + \sigma_L^2)^2} - \frac{(\Delta x_m)^2 \Delta t^2}{2(D\Delta t + \sigma_L^2)^3}$$

The Fisher information $I(D)$ from a single displacement measurement is the negative expected value of the second derivative:

$$I(D) = -E\left[\frac{\partial^2}{\partial D^2}\log f\right]$$

Since $E\left[(\Delta x_m)^2\right] = 2(D\Delta t + \sigma_L^2)$, we have:

$$I(D) = -\left(\frac{\Delta t^2}{2(D\Delta t + \sigma_L^2)^2} - \frac{2(D\Delta t + \sigma_L^2)\Delta t^2}{2(D\Delta t + \sigma_L^2)^3}\right) = \frac{\Delta t^2}{2(D\Delta t + \sigma_L^2)^2}$$

For n independent measurements:

$$I_{\text{total}}(D) = n \times I(D) = \frac{n\Delta t^2}{2(D\Delta t + \sigma_L^2)^2}$$

10.2.2 Cramér-Rao Lower Bound

The CRLB for diffusivity D, considering the localization error, is:

$$\text{Var}(\hat{D}) \geq \frac{1}{I_{\text{total}}(D)} = \frac{2(D\Delta t + \sigma_L^2)^2}{n\Delta t^2}$$

Simplifying:

$$\text{Var}(\hat{D}) \geq \frac{2}{n}\left(\frac{D\Delta t + \sigma_L^2}{\Delta t}\right)^2$$

The localization precision σ_L is related to the measurement noise σ and the experimental parameters:

$$\sigma_L = \frac{\sigma}{\sqrt{cI_0L|\alpha_t|^2\frac{k^3}{4}}}$$

Substituting σ_L into the CRLB expression:

$$\sigma_L^2 = \left(\frac{\sigma}{\sqrt{cI_0L|\alpha_t|^2\frac{k^3}{4}}}\right)^2 = \frac{4\sigma^2}{cI_0L|\alpha_t|^2k^3}$$

Thus, the CRLB becomes:

$$\text{Var}(\hat{D}) \geq \frac{2}{n}\left(\frac{D\Delta t + \frac{4\sigma^2}{cI_0L|\alpha_t|^2k^3}}{\Delta t}\right)^2$$

Simplify the numerator inside the parentheses:

$$D\Delta t + \frac{4\sigma^2}{cI_0L|\alpha_t|^2k^3} = \Delta t\left(D + \frac{4\sigma^2}{cI_0L|\alpha_t|^2k^3\Delta t}\right)$$

Therefore, the CRLB simplifies to:

$$\text{Var}(\hat{D}) \geq \frac{2}{n}\left(D + \frac{4\sigma^2}{cI_0L|\alpha_t|^2k^3\Delta t}\right)^2$$

10.2.3 Final CRLB Expression for Diffusivity in NSM

The modified CRLB for diffusivity D in NSM, incorporating the localization precision, is:

$$\boxed{\mathrm{Var}(\hat{D}) \geq \frac{2}{n}\left(D + \frac{4\sigma^2}{cI_0L|\alpha_t|^2k^3\Delta t}\right)^2}$$

The term $\frac{4\sigma^2}{cI_0L|\alpha_t|^2k^3\Delta t}$ represents the influence of localization error on the variance of $\hat{D}$. Reducing measurement noise σ or increasing the detected signal improves localization precision, thus reducing this term. Increasing Δt reduces the impact of localization error relative to diffusion, enhancing estimation precision. The variance decreases inversely with the number of measurements, emphasizing the benefit of collecting more data.

In the absence of localization error ($\sigma_L^2 = 0$), the CRLB reduces to:

$$\mathrm{Var}(\hat{D}) \geq \frac{2D^2}{n}$$

which matches the standard result derived without considering localization error.

References

[1] Piliarik, M., Sandoghdar, V. Direct optical sensing of single unlabelled proteins and super-resolution imaging of their binding sites. *Nature Communications* **5**, 4495 (2014).

[2] Špačková, B., Klein Moberg, H., Fritzsche, J., Tenghamn, J., Sjösten, G., Šípová-Jungová, H., Albinsson, D., Lubart, Q., van Leeuwen, D., Westerlund, F., Midtvedt, D., Esbjörner, E. K., Käll, M., Volpe, G., Langhammer, C. Label-free nanofluidic scattering microscopy of size and mass of single diffusing molecules and nanoparticles. *Nature Methods* **19**, 751–758 (2022).

[3] Dechadilok, P., Deen, W. M. Hindrance factors for diffusion and convection in pores. *Industrial & engineering chemistry research* **45**, 6953–6959 (2006).

[4] Weiss, K., Khoshgoftaar, T. M., Wang, D. A survey of transfer learning. *Journal of Big Data* **3**, 9 (2016).

[5] Zeiler, M. D., Fergus, R. Visualizing and understanding convolutional networks (2013).

[6] Csikszentmihalyi, M. *Flow: The Psychology of Optimal Experience* (1990).

[7] Zeiler, M. D., Fergus, R. Fleet, D., Pajdla, T., Schiele, B., Tuytelaars, T. (eds), *Visualizing and understanding convolutional networks. Computer Vision – ECCV*

2014 (Fleet, D., Pajdla, T., Schiele, B., Tuytelaars, T. eds), 818–833 (Springer International Publishing, Cham, 2014).

[8] Bengio, Y., Louradour, J., Collobert, R., Weston, J. *Curriculum learning. Proceedings of the 26th Annual International Conference on Machine Learning*, ICML '09, 41–48 (Association for Computing Machinery, New York, NY, USA, 2009).

[9] Midtvedt, B., Helgadottir, S., Argun, A., Pineda, J., Midtvedt, D., Volpe, G. Quantitative digital microscopy with deep learning. *Applied Physics Reviews* **8**, 011310 (2021).

[10] Kingma, D. P., Ba, J. Adam: A method for stochastic optimization. *CoRR* **abs/1412.6980** (2014).

[11] Ronneberger, O., P.Fischer, Brox, T. *U-net: Convolutional networks for biomedical image segmentation. Medical Image Computing and Computer-Assisted Intervention (MICCAI)*, Vol. 9351 of *LNCS*, 234–241 (Springer, 2015).

[12] Roeder, L. Netron. https://github.com/lutzroeder/netron (2020).

[13] Rivenson, Y., Liu, T., Wei, Z., Zhang, Y., de Haan, K., Ozcan, A. Phasestain: the digital staining of label-free quantitative phase microscopy images using deep learning. *Light: Science & Applications* **8**, 23 (2019).

[14] Jensen, S. S., Jensen, H., Cornett, C., Møller, E. H., Østergaard, J. Insulin diffusion and self-association characterized by real-time UV imaging and taylor dispersion analysis. *J Pharm Biomed Anal* **92**, 203–210 (2014).

[15] Virtanen, P., Gommers, R., Oliphant, T. E., Haberland, M., Reddy, T., Cournapeau, D., Burovski, E., Peterson, P., Weckesser, W., Bright, J., van der Walt, S. J., Brett, M., Wilson, J., Millman, K. J., Mayorov, N., Nelson, A. R. J., Jones, E., Kern, R., Larson, E., Carey, C. J., Polat, İ., Feng, Y., Moore, E. W., VanderPlas, J., Laxalde, D., Perktold, J., Cimrman, R., Henriksen, I., Quintero, E. A., Harris, C. R., Archibald, A. M., Ribeiro, A. H., Pedregosa, F., van Mulbregt, P., SciPy 1.0 Contributors. SciPy 1.0: Fundamental Algorithms for Scientific Computing in Python. *Nature Methods* **17**, 261–272 (2020).

[16] Harris, C. R., Millman, K. J., van der Walt, S. J., Gommers, R., Virtanen, P., Cournapeau, D., Wieser, E., Taylor, J., Berg, S., Smith, N. J., Kern, R., Picus, M., Hoyer, S., van Kerkwijk, M. H., Brett, M., Haldane, A., del Río, J. F., Wiebe, M., Peterson, P., Gérard-Marchant, P., Sheppard, K., Reddy, T., Weckesser, W., Abbasi, H., Gohlke, C., Oliphant, T. E. Array programming with NumPy. *Nature* **585**, 357–362 (2020).

[17] Virtanen, P., Gommers, R., Oliphant, T. E., Haberland, M., Reddy, T., Cournapeau, D., Burovski, E., Peterson, P., Weckesser, W., Bright, J., á others. SciPy

1.0: fundamental algorithms for scientific computing in python. *Nat. Methods* **17**, 261–272 (2020).

[18] Lin, J. Divergence measures based on the shannon entropy. *IEEE Transactions on Information Theory* **37**, 145–151 (1991).

[19] Kullback, S., Leibler, R. A. On information and sufficiency. *Annals of Mathematical Statistics* **22**, 79–86 (1951).

[20] Villani, C. *Optimal Transport: Old and New* Vol. 338 of *Grundlehren der mathematischen Wissenschaften* (Springer, 2009).

[21] Kolmogorov, A. Sulla determinazione empirica di una legge di distribuzione. *Giornale dell'Istituto Italiano degli Attuari* **4**, 83–91 (1933).

[22] Smirnov, N. Table for estimating the goodness of fit of empirical distributions. *Annals of Mathematical Statistics* **19**, 279–281 (1948).

[23] Heusel, M., Ramsauer, H., Unterthiner, T., Nessler, B., Hochreiter, S. *Gans trained by a two time-scale update rule converge to a local nash equilibrium. Advances in Neural Information Processing Systems*, Vol. 30 (2017).

[24] Gretton, A., Borgwardt, K., Rasch, M., Schölkopf, B., Smola, A. A kernel two-sample test. *Journal of Machine Learning Research* **13**, 723–773 (2012).

[25] Cramér, H. *Mathematical Methods of Statistics* (Princeton University Press, 1946).

[26] Rao, C. R. Information and the accuracy attainable in the estimation of statistical parameters. *Bulletin of the Calcutta Mathematical Society* **37**, 81–91 (1945).

[27] Kay, S. M. *Fundamentals of Statistical Signal Processing, Volume I: Estimation Theory* (Prentice Hall, 1993).

[28] Feller, W. *An Introduction to Probability Theory and Its Applications* 3rd edn, Vol. 1 (John Wiley & Sons, 1968).

[29] Lehmann, E. L., Casella, G. *Theory of Point Estimation* 2nd edn (Springer, 1998).
